# A leading-edge scenario in the phylogeography and evolutionary history of East Asian insular *Taxus* in Taiwan and the Philippines

**DOI:** 10.3389/fgene.2024.1372309

**Published:** 2024-05-02

**Authors:** Hao-Chih Kuo, Travis Schoneman, Lian-Ming Gao, William Sm. Gruezo, Victor B. Amoroso, Yang Yang, Kuo-Cheng Yang, Ching-Te Chien, Michael Möller, Chun-Neng Wang

**Affiliations:** ^1^ Institute of Ecology and Evolutionary Biology, National Taiwan University, Taipei, Taiwan; ^2^ Key Laboratory for Plant Diversity and Biogeography of East Asia, Kunming Institute of Botany, Chinese Academy of Sciences, Kunming, Yunnan, China; ^3^ Plant Biology Division, College of Arts and Sciences, Institute of Biological Sciences, University of the Philippines at Los Baños, Laguna, Philippines; ^4^ Center for Biodiversity Research and Extension in Mindanao (CEBREM), Central Mindanao University, Mindanao, Philippines; ^5^ Tainan District Agricultural Research and Extension Station, Ministry of Agriculture, Tainan, Taiwan; ^6^ General Education Center, Providence University, Taichung, Taiwan; ^7^ Botanical Garden Division, Taiwan Forestry Research Institute, Taipei, Taiwan; ^8^ Royal Botanic Garden Edinburgh, Edinburgh, United Kingdom; ^9^ Department of Life Science, National Taiwan University, Taipei, Taiwan

**Keywords:** introgression, long-distance colonization, post-split migration, seed dispersal, *Taxus mairei*, *Taxus phytonii*, yew trees

## Abstract

The cool temperate origin of gymnosperm *Taxus* species in East Asia is specifically diverse and widespread. Certain lineages have managed to extend their distribution further south to subtropical and tropical islands such as Taiwan and the Philippines. To address questions including whether these insular lineages, recently identified as *T. phytonii*, have become genetically distinct from each other and from their continental relatives, and when and how they colonized their residing islands, we sampled over 11 populations, covering 179 *Taxus* individuals from Taiwan and the Philippines. Using four cpDNA and one nuclear marker, we showed in population genetic and genealogical analyses that the two insular lineages were genetically distinct from each other and also from other continental *Taxus* and that they represented each other’s closest relative. Estimated with the coalescent-based multi-type tree (MTT) analyses, we inferred an origin of Taiwanese *T. phytonii* more ancient than 2.49 Mya and that of Philippine *T. phytonii* more ancient than 1.08 Mya. In addition, the divergence demographic history revealed by both MTT and isolation with migration (IM) analyses indicated the presence of recent post-split migrations from a continental taxon, *T. mairei*, to Taiwanese *T. phytonii*, as well as from Taiwanese *T. phytonii* to Philippine *T. phytonii*. Overall, this study suggests Taiwan as a stepping stone through which the temperate-origin yew trees can extend their distributions to tropical regions such as the Philippines.

## Introduction

The eastern and southeastern parts of Asia are remarkably rich in gymnosperm diversity (Liu et al., 2022). Many are endemic there, such as the so-called ‘relicts’ and monotypic *Ginkgo* L. (Ginkgoaceae), *Taiwania* Hayata and *Metasequoia* Hu & W.C.Cheng (both Cupressaceae), and *Pseudotaxus* W.C.Cheng (Taxaceae), that remain in only a few isolated locations, while related species have all become extinct around the world (Tang et al., 2018; Kou et al., 2020; Liu et al., 2022). Other relic gymnosperms residing in East Asia, particularly evergreen gymnosperms, however, are widely distributed and have greatly diversified (López-Pujol et al., 2011; Tang et al., 2018; Liu et al., 2022). *Taxus*, commonly referred to as the yew tree, is a gymnosperm genus of the order Cupressales which is widely distributed across the temperate Northern Hemisphere region and reaching the tropics in Malesia (Yang et al., 2022). Regions in the contemporary subtropical mainland Asia and Southeast Asia are important refugia for relic species that evolved before the late Tertiary and early Quaternary Periods because continental glaciers did not reach this area during glacial maxima (Axelrod et al., 1998). Glacial–interglacial cycles will have allowed expansions and contractions of populations during cooler and warmer periods, respectively, allowing and severing gene flow in these areas that resulted in the formation of 11 *Taxus* lineages in East and Southeast Asia by the Late Miocene, with another lineage covering entire Europe (Liu et al., 2011; Liu et al., 2018; Fu et al., 2019).

Due to the intricate topography of East Asia, more recent glacial–interglacial cycles in the Pliocene/Pleistocene may have affected gene flow in Asian yew populations more pronounced (Zhang et al., 2009; Jia et al., 2022). Earlier analyses of the population genetics of the East Asian *Taxus* species demonstrated a notable level of genetic differentiation across various regions, particularly along separated mountain ranges, specifically among populations of the Himalaya–Hengduan Mts., Qinling Mts., and Mt. Emei and on the east and west sides of the Wuyi–Nanling mountain range (Gao et al., 2007; Zhang et al., 2009; Möller et al., 2013; Liu et al., 2018; Qin et al., 2023).

The distribution of East Asian *Taxus* species also extends to the continental island of Taiwan and the tropical oceanic islands of the Philippines and Indonesia (Moller et al., 2020; Kamal et al., 2023). However, phylogeographic and genetic diversity studies on island Sumatran yew suggested their genetic identity belongs to *T. mairei* and there exists no genetic variation among wild populations, indicating they are recent migrants (Rachmat et al., 2016; Kamal et al., 2023). On the other hand, all other phylogeographic analyses on the Northern Hemisphere *Taxus* using both cpDNA and nuclear markers, although with very limited individual samples, suggest Taiwanese and Philippine yews, diverged as very distinct island-migrating lineages sister to all other southern China *Taxus*, *T. mairei*, and *T. calcicola* (Gao et al., 2007; Zhang et al., 2009; Liu et al., 2011; Liu et al., 2018; Fu et al., 2019; Möller et al., 2020). These Taiwanese and Philippine *Taxus* may have followed a similar colonization history.

Although the phylogeography and genetic differentiation of *Taxus* species in continental Asia have been relatively well-studied, much less is known for the migration history and divergence of *Taxus* species occurring on islands such as those in Taiwan and the Philippines. Based on only one population sample, the cpDNA or ISSR haplotypes of Taiwanese *Taxus* were found closely linked to *Taxus mairei* haplotypes in mainland Asia (Gao et al., 2007; Zhang et al., 2009). Interestingly, with only three individuals of Philippine *Taxus* being included, barcoding analyses suggested that the Philippine yew was very closely related to the Taiwanese yew (Liu et al., 2011; Liu et al., 2018; Fu et al., 2019). *Taxus* populations from Taiwan and the Philippines have, therefore, been suggested to represent *T. phytonii* recently (Liu et al., 2018). Molecular clock estimation based on cpDNA markers suggested that colonization of the Taiwanese yew from mainland China was approximately 3.5 million years ago (Mya) likely during a glacial maximum (95% HDP: 1.0–8.3 Mya) (Möller et al., 2020). This implies that these island yews are not young and recent migrants but maybe considerably old.

Although most of the cool temperate origin East Asia *Taxus* species are distributed in the middle of the genus’ natural range in mainland Asia, the insular *T. phytonii* populations in Taiwan and the Philippines, therefore, exist as the leading edge for *Taxus* species, living at their ecological limits, that is, the subtropical and tropical habitats. Because these insular *T. phytonii* populations are found only surviving at high mountains as isolated interglacial patches, these leading-edge island yews may contain the genetic signature of Pleistocene glacial refugia as important hotspots for preserving the *Taxus* genetic diversity (Myers et al., 2000; Tang et al., 2018). In comparison to the central populations, the leading edge is important to understand how the founder populations were established and whether these populations confer climate-adaptive traits (Petit et al., 2003; Hampe and Petit, 2005). In particular, the colonized individuals of these subtropical/tropical leading-edge populations might contain heat stress-adapted genetic alleles, which could be very valuable for the conservation of genetic diversity in *Taxus* species. Genetic identification on these island yew populations is also crucial, providing they are the final missing pieces of the DNA barcode reference library, while all other mainland Asia and global *Taxus* species have been investigated (Liu et al., 2011; Liu et al., 2018).

Realizing the colonization history of these insular *T. phytonii* is equally important as this helps unravel the possible routes of usual overseas long-distance dispersal of *Taxus* migration (e.g., Taiwan Strait >130 km wide; Luzon Strait >250 km). For the dispersal of *Taxus* from continental Asia to Taiwan, it might be explained by migration during glacial maxima over land bridges connecting mainland China and Taiwan (Ota, 1998). For the migration to the Philippines, this implies not only the occurrence of long-distance dispersal of *Taxus* species but also hypotheses such as tectonic movement, stepping stone islands, and land bridges (Pleistocene sea level fall during glacial maximum) have been suggested for the formation of Philippines’ flora (Hughes et al., 2015). *Taxus* seeds are surrounded by red, fleshy arils, which attract birds and are thus well-suited for endozoochorous long-distance dispersal (Wilson et al., 1996; Thomas and Polwart, 2003). Thus, *Taxus* populations in continental Asia and southeastern Asian islands might have been subjected to migrations and vicariance events, as influenced by climate fluctuations (Jia et al., 2022). Inferring the effects of these events from the molecular evolutionary rates could help reveal the phylogeographic history of insular *Taxus* in East Asia.

In this study, we have assembled 179 individuals in total from nine and two populations from Taiwan and the Philippines, respectively. Our main aims are, therefore, as follows: (1) to reconstruct phylogenetic trees and haplotype networks using both cpDNA and nuclear markers to infer the genealogical relationships of insular *T. phytonii* and their sister lineages in East Asia; (2) to perform gene flow direction analyses to deduce the origin and the possible colonization routes of insular *T. phytonii*; and (3) to calculate the divergence time of insular *T. phytonii* to interpret the post-divergence migration events between *T. phytonii* and East Asian *Taxus*. The results shall extend our understanding of the evolutionary history of these insular *Taxus* populations and whether they retain the refugial genetic signature for considering conservation.

## Materials and methods

### Tissue sampling and molecular markers

**FIGURE 1:**
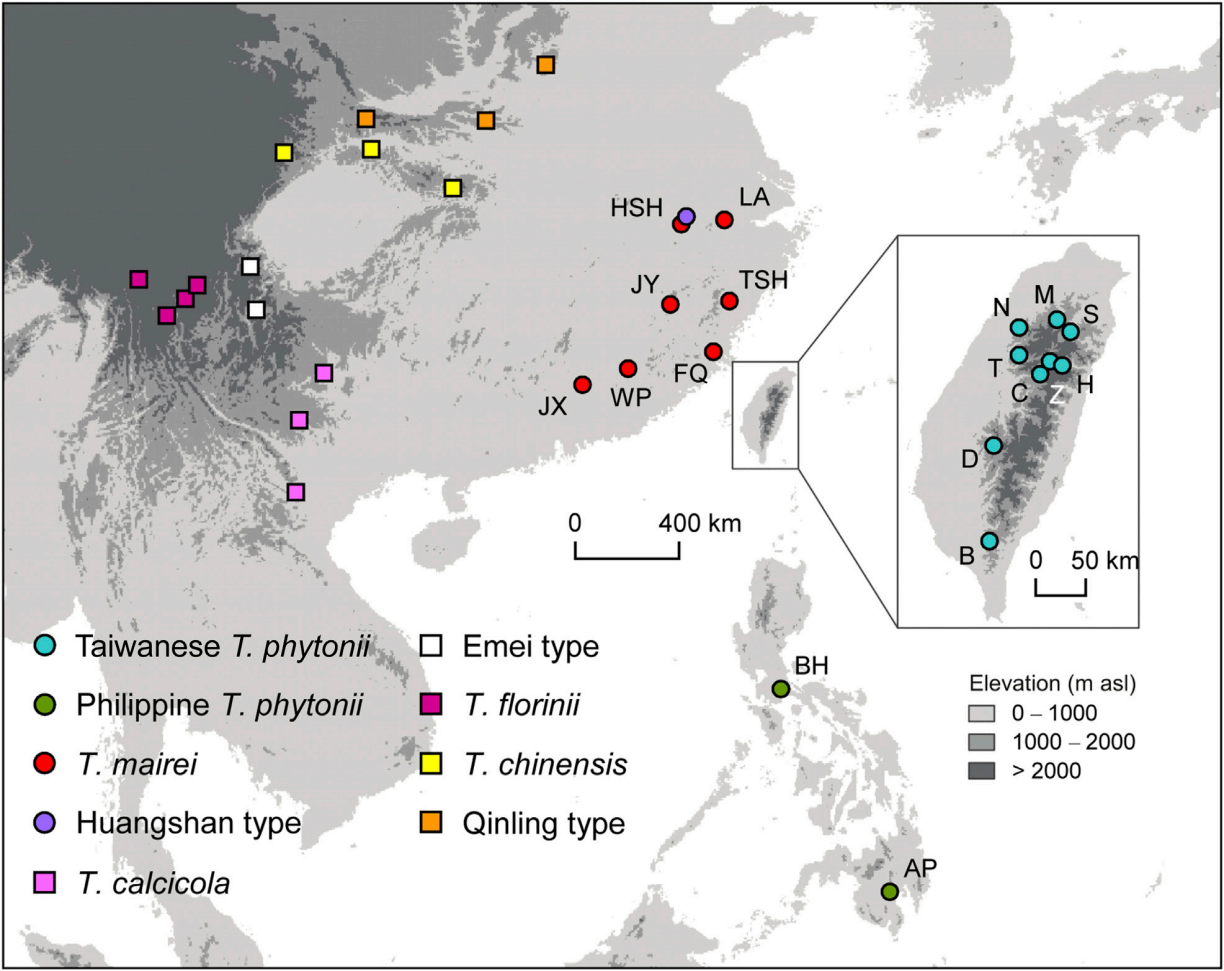
Map showing sampling sites of all *Taxus* taxa included in the study. Circles and squares denote populations which have DNA sequences newly generated in this study or obtained from public databases, respectively. Codes for taxa marked by circles are shown in Table 1.

Young, healthy leaves were collected and quickly dried in silica gel from 141 and 38 individual trees of *T. phytonii* (as *T. sumatrana* in Liu et al., 2011) from Taiwan and the Philippines, respectively. Serving as comparative materials, samples were also collected from 105 *T. mairei* (=*T. wallichiana* var. *mairei* in Gao et al., 2007), a taxon closely allied to *T. phytonii* both geographically and phylogenetically (Gao et al., 2007; Liu et al., 2011; Fu et al., 2019). We also included five other *Taxus* samples from higher elevations (>1,500 m a.s.l.) of Huangshan, China; we followed Möller et al. (2020) to refer to this taxon as the Huangshan type. Given the close geographic proximity of Huangshan to Taiwan, genetic exchanges may also have occurred between these places during past glacial maxima. For yew taxa described above, sampling was carried out in a total of 19 populations (Figure 1; Table 1).

**TABLE 1:** Sampling of four *Taxus* taxa. Measures of genetic variability based on chloroplast DNA or the nuclear ITS markers are provided. Based on the ITS variability, samples from Mt. Apo, Mindanao, show a significant deviation from the Hardy–Weinberg equilibrium (F_IS_ shown in bold).

				Chloroplast DNA^a^			ITS			
Taxon	Site (code)	Longitude/latitude	n	Hd	π	θ_W_	Hd	π	θ_W_	F_IS_
Taiwanese *T. phytonii*	Anmashan, Taichung (T)	121° 01'E/24° 16′N	28	0.50	0.42	0.22	0.59	1.48	1.55	−0.03
	Nanzhuang, Miaoli (N)	121° 01′E/24° 31′N	16	0	0	0	0.61	2.23	1.78	−0.06
	Smangus, Hsinchu (M)	121° 21′E/24° 35′N	16	0.43	0.60	1.03	0.34	1.42	1.78	−0.05
	Siji, Yilan (S)	121° 28′E/24° 29′N	18	0.11	0.09	0.25	0.48	0.83	0.65	−0.18
	Bilu Shenmu, Hualien (H)	121° 24′E/24° 11′N	15	0.51	0.76	1.19	0.43	1.44	2.03	−0.08
	Zhongheng Highway, Hualien (Z)	121° 17′E/24° 13′N	14	0.14	0.12	0.27	0.30	0.54	0.46	0.30
	Cuifeng, Nantou (C)	121° 12′E/24° 06′N	15	0.25	0.21	0.26	0.62	1.59	1.80	−0.07
	Dabang, Chiayi (D)	120° 47′E/23° 28′N	15	0.13	0.11	0.26	0.55	1.25	1.58	−0.22
	Mt. Beidawu, Pingtung (B)	120° 45′E/22° 37′N	4	0.50	0.42	0.46	0.54	0.96	0.69	−0.50
	Total	-	141	0.52	0.51	0.70	0.57	1.48	1.87	-
Philippine *T. phytonii*	Mt. Banahaw, Luzon (BH)	121° 28′E/14° 03′N	18	0	0	0	0.21	0.38	0.84	−0.06
	Mt. Apo, Mindanao (AP)	125° 16′E/07° 00'N'	20	0.19	0.08	0.12	0.51	1.79	0.82	**−0.90**
	Total	-	38	0.10	0.04	0.10	0.46	1.48	1.25	-
*T. mairei*	Huangshan, Anhui, China (HSH)	118° 12′E/30° 05′N	12	0.30	0.13	0.14	0.50	1.09	1.85	−0.18
	Lin’an, Zhejiang, China (LA)	119° 30′E/30° 19′N	15	0.42	0.18	0.13	0.78	2.47	4.81	0.15
	Jianyang, Fujian, China (JY)	117° 39′E/27° 25′N	15	0.51	0.22	0.13	0.46	0.82	1.32	0.43
	Taishun, Zhejiang, China (TSH)	119° 41′E/27° 31′N	15	0.42	0.18	0.13	0.78	1.70	2.20	0.15
	Jiulianshan, Jiangxi, China (JX)	114° 34′E/24° 37′N	15	0.34	0.15	0.13	0.45	0.91	1.53	−0.19
	Wuping, Fujian, China (WP)	116° 10′E/25° 11′N	15	0.34	0.15	0.13	0.54	0.62	0.66	−0.24
	Fuqing, Fujian, China (FQ)	119° 08′E/25° 46′N	18	0.11	0.05	0.12	0.71	1.41	1.46	−0.02
	Total	-	105	0.48	0.21	0.08	0.66	1.31	4.27	-
Huangshan type	Huangshan, Anhui, China (HSH)	118° 12′E/30° 05′N	5	0	0	0	0.91	3.94	4.32	−0.11

n, number of individuals; Hd, haplotype diversity; π, nucleotide diversity per nucleotide site; θW, Watterson’s (1975) theta per nucleotide site; FIS, inbreeding coefficient. Values of π and θW are shown as raw values multiplied by 1,000.

^a^ Contains trnL-trnF, trnH-psbA, petG-trnP, and atpI-atpH.

DNA was isolated from silica gel-dried leaves following a standard CTAB procedure (Doyle, 1991), with modifications: buffer consisting of 4% CTAB (Amresco, United States of America) (instead of 2%) and 200 mM Tris-HCl (J.T. Baker, United States of America) (instead of 100 mM); a pinch of PVPP (Sigma, United States of America) was freshly added before adding the CTAB buffer to the ground tissue samples. Among the four cp DNA markers (i.e., *trn*L*-*F, *trn*H*-psb*A, *pet*G*-trn*P, and *atp*I*-*H) plus one nuclear marker (internal transcribed spacer of the 18S-5.8S-26S ribosomal cistron, and ITS) were selected following previous literature reports. Three of these markers (*trn*L*-*F, *trn*H*-psb*A, and ITS) were selected following previous literature reports in an attempt to join sequencing efforts, allowing the sample comparison of *T. phytonii* to other East Asian *Taxus*. In trying to add up more sequence polymorphism for resolving *Taxus* phylogeography, two additional chloroplast markers *pet*G*-trn*P and *atp*I*-*H were used because they have been found useful to reconstruct gymnosperm phylogeny (Chou et al., 2011).

Marker amplification protocols and procedures are detailed in Supplementary Appendix S1 (with used primers listed in Supplementary Table S1). For ITS, 169 of the 289 samples (59%) across the four analyzed taxa had heterozygous single-nucleotide positions and/or indels (shown as double peaks in electropherograms at or after specific nucleotide sites), and for each such case, various approaches were undertaken to uncover the two haplotypes for each individual (detailed in Supplementary Appendix S1). Marker-wise sequence alignments were carried out with muscle v3.8.31 (Edgar, 2004) implemented in MEGA 11 (Tamura et al., 2021). The recombination-free inheritance nature of cpDNA (Birky, 2001) would render the cp genome as a whole, behaving as a single locus, although certain cp regions in yews have reported the existence of isomeric forms. Consequently, we concatenated the four cp markers by individuals in all subsequent analyses.

### Genetic variability and population clustering

Genetic variability within populations (defined by sampling sites) of the four focal *Taxus* taxa was assessed with measures including the haplotype diversity (Hd, Nei and Tajima, 1981), nucleotide diversity (π, Nei and Li, 1979), and Watterson’s (1975) theta (θ_W_), all calculated in DnaSP v.6.12.03 (Rozas et al., 2017). The within-individual ITS variation in more than half of the analyzed individuals might indicate that this marker is not completely homogenized as is typically expected (Baldwin et al., 1995). We, thus, assessed conformity of ITS variation to the Hardy–Weinberg equilibrium (HWE), a status presumably achieved when the marker was biparentally inherited as a single locus. Alternatively, a deviation from the HWE in the form of heterozygote excess is expected in cases where incomplete concerted evolution only homogenized intra-locus variation but not that across different loci within the genome (Karvonen and Savolainen, 1993). We treated different ITS haplotypes as different alleles, with which Weir and Cockerham’s (1984) F_IS_ was calculated as a measure of HWE deviation levels (zero for no deviation, and positive and negative values for heterozygote excess and deficiency, respectively). A Markov chain algorithm (Guo and Thompson, 1992) was used to evaluate significance of deviation from the HWE, with Bonferroni multiple-testing corrections applied to control a table-wide error rate of <0.05. These examinations on population-wise HWE were carried out in Genepop v.4.2 (Raymond and Rousset, 1995; Rousset, 2008).

We examined further whether variation among populations of the same taxon was smaller than that among different taxa. To this end, we quantified genetic differentiation between pairwise populations using Φ_ST_ (Excoffier et al., 1992). We then constructed cp and ITS neighbor-joining trees (NJ, Saitou and Nei, 1987; Saitou and Nei, 1987) based on Φ_ST_ values and examined whether populations were clustered according to the taxon identity. We further conducted Φ-statistic-based analyses of molecular variance (AMOVA, Excoffier et al., 1992) to quantify the genetic variation partitioned along a hierarchical structure of grouping ranks from among taxa to within populations (or within individuals for ITS). Permutation tests were undertaken to assess the significance of partitioned variation at different ranks (2,000 permutations for each). Both Φ_ST_ calculations and AMOVA were carried out in Arlequin v.3.5.2.2 (Excoffier and Lischer, 2010), while NJ tree constructions were carried out with the ape and phytools R packages (Revell, 2012; Paradis and Schliep, 2019).

### Haplotype tree and network reconstructions

Cp and ITS haplotype trees and networks were reconstructed for investigating the genealogical relationships of the four focal taxa with each other and with the other East Asian *Taxus*. For this, cp sequences from Fu et al. (2019) and ITS sequences from Liu et al. (2011) for East Asian taxa (Supplementary Tables S2, S3 of Supplementary Appendix S1) were pooled with our newly acquired data for combined analyses. The Bayesian calculation implemented in MrBayes v.3.2.7 (Ronquist et al., 2012) was used for haplotype tree reconstructions. To root the trees, two Taxaceae species, *Pseudotaxus chienii* and *Cephalotaxus manni*, were included as the outgroup (Supplementary Tables S2, S3 of Supplementary Appendix S1). The GTR + G and the HKY + G nucleotide substitution models (Hasegawa et al., 1985; Tavaré, 1986; Yang, 1994) were specified for the cp and the ITS datasets, respectively, both of which were selected using jModelTest v.2.1.3 (Darriba et al., 2012). For each marker, two replicate runs of Metropolis-coupled Markov chain Monte Carlo (MCMC) were performed, with the incremental heating scheme applied to improve the exploring of the parameter space. The MCMC was run for 1,000,000 iterations (every 500th iteration for a sample), discarding the initial 100,000 iterations as burn-in.

The haplotype networks were built using the statistical parsimony (SP) algorithm (Templeton et al., 1992) implemented in TCS v.1.21 (Clement et al., 2000). In each analysis, a *p*-value of >0.95 for parsimonious connections was adopted to join haplotypes into a single network; otherwise, separate networks were built. To incorporated indel events into genealogical reconstructions, we applied Simmons and Ochoterena (2000) ‘simple indel coding’ method implemented in FastGap v.1.2 (Borchsenius, 2009).

### Post-split migration detection

We identified from the reconstructed haplotype networks shares of lineages between *T. mairei* and *T. phytonii* and between the two *T. phytonii* gene pools from Taiwan and the Philippines. Such lineage sharing could stem from migrations between divergent taxa (called ‘post-split migrations’ for they occur after the taxa started to diverge) or, alternatively, could represent incomplete lineage sorting events, a legacy of lineages present in the common ancestor of these taxa. To distinguish between these two scenarios, we performed a Bayesian analysis which was based on the isolation with migration (IM) model implemented in IMa2 (Hey and Nielsen, 2007; Hey, 2010) to assess the presence of post-split migrations.

Applied to *T. mairei* and *T. phytonii* from Taiwan and the Philippines, respectively, we specified the topology of taxon divergence as [(Taiwanese *T. phytonii*, Philippine *T. phytonii*), *T. mairei*] based on our haplotype network reconstruction results and from aforementioned publications (Liu et al., 2018; Fu et al., 2019; Möller et al., 2020). We specified the HKY nucleotide substitution model (Hasegawa et al., 1985) implemented in IMa2 for both cp and ITS sequence sets. CpDNA is haploid and, in *Taxus*, paternally inherited (Anderson and Owens, 1999), and our HWE tests suggested ITS, following a single-locus biparental inheritance mode in the focal taxa (see Results). Accordingly, we set inheritance scalars of cpDNA and ITS to 0.25 and 1, respectively. Truncated uniform priors were implemented in IMa2 for demographic parameters including the rescaled post-split migration rates (*m*), the composite parameters for effective population sizes (*q*), and the composite parameters for split times between taxa (*t*). Upper bounds of *m*, *q*, and *t* priors were set to 1.5, 9, and 6, respectively, which were derived under synthetic considerations over the manufacturer’s suggestions (Hey, 2011) and results from pilot exploratory runs (detailed in Settings for demographic priors and Supplementary Table S4 of Supplementary Appendix S1). A Bayesian calculation was carried out with three independent runs of Metropolis-coupled MCMC, using a geometric heating scheme with 40 heated chains and heating parameters *ha* = 0.95 and *hb* = 0.80 for each run. The MCMC was initially run for 3,00,000 iterations for burn-in, followed by additional one million iterations for sampling (every 100th iteration for a sample). To convert resultant *q* and *t* estimates to effective population sizes (N_e_) and divergence times in absolute years, respectively, we specified locus-wide mutation rates of cpDNA and ITS as 1.67E-6 and 1.24E-6 per year, respectively (see Clock rates of chloroplast DNA and ITS for *Taxus* and Supplementary Table S5 of Supplementary Appendix S1) and a *Taxus* generation time of 25 years (Wang et al., 2006). Finally, the presence of post-split migrations between taxon pairs was evaluated using likelihood ratio tests proposed by Nielsen and Wakeley (2001) based on estimated population migration rates 2N_e_ × M (M for the proportion of migrants per population per generation).

### Inference of the colonization direction

We performed the cp- and the ITS-based multi-type tree (MTT) analyses to investigate *Taxus* migration events connecting Taiwan and the Philippines. To infer colonization history, we performed Bayesian analyses based on the single-locus MTT) (Vaughan et al., 2014) coalescent models to trace the location (either Taiwan or Philippines) of individual lineages and their switching between locations along the gene tree. Importantly, De Maio et al. (2015) demonstrated that MTT provided unbiased inferences under highly uneven sample sizes associated with different locations, as in our case. We aimed to infer emigrations from one of the two insular *Taxus* regions that first time colonized the other one, which resulted in the *Taxus* origin in the latter region. The entry sequences of our MTT analyses were those constituting the insular haplogroup.

The MTT analyses were performed in BEAST v.2.7.6 (Bouckaert et al., 2019). For both the cp- and ITS-based analyses, we specified nucleotide substitutions, following the F81 model (Felsenstein, 1981), selected using jModelTest v.2.1.3 (Darriba et al., 2012). For time calibrations, we specified the following lognormal clock rate priors: log (mean) = −4.93 and log (standard deviation) = 0.27 for cpDNA; log (mean) = −4.50 and log (standard deviation) = 0.27 for ITS (see Clock rates of chloroplast DNA and ITS for *Taxus* and Supplementary Table S5 of Supplementary Appendix S1 for details). Truncated uniform priors each with an upper bound = 1 and lognormal priors each with a log (mean) = 0 and log (standard deviation) = 4 were used for population size and migration rate parameters, respectively (see Settings for demographic priors of Supplementary Appendix S1 for setting rationales). For each of the cp- and ITS-based analyses, we constructed two independent MCMC that each was run for 100 million iterations (every 100,000^th^ iteration for a sample) with the initial 10% discarded as burn-in. MCMC convergence between the two replicate runs was examined with Tracer v.1.7.2 (Rambaut et al., 2018), and the results were combined to give an effective sample size (ESS) of >200 per parameter.

## Results

### Genetic variability and population clustering of the four focus yew taxa

From the four focus yew taxa, namely, Taiwanese *T. phytonii*, Philippine *T. phytonii*, *T. mairei*, and Huangshan type, we identified eight cp haplotypes based on the concatenated cp sequences (see Supplementary Figure S1A of Supplementary Appendix S2 for source populations of these haplotypes). The populations of the four taxa showed modest cp genetic variability, with haplotype diversity (Hd), nucleotide diversity (π, per nucleotide site), and Watterson’s theta (θ_W_, per nucleotide site) being 0–0.51, 0–0.00076, and 0–0.00119, respectively (Table 1). We identified from the four taxa totally 32 ITS haplotypes (source populations shown in Supplementary Figure S1B of Supplementary Appendix S2). ITS-based Hd, π, and θ_W_ values were between 0.21 and 0.91, 0.00038 and 0.00394, and 0.00046 and 0.00481, respectively (Table 1), which were higher than the corresponding cp-based values. In general, populations of the Philippine *T. phytonii* bore lower genetic variability compared to those of the other three taxa (Table 1).

All *Taxus* populations examined had ITS variation conforming to HWE except the Philippine *T. phytonii* population on Mt. Apo, Mindanao (AP), which showed significant excess of heterozygotes (F_IS_ = −0.9, Markov chain estimation *p* = 0 after Bonferroni correction; Table 1). Provided that most populations conformed to the HWE and did not show uniformly positive or negative F_IS_ values (refuting that ITS variation was subject to specific systematic biases), we assumed ITS to follow a single-locus biparental inheritance mode in downstream analyses.

**FIGURE 2:**
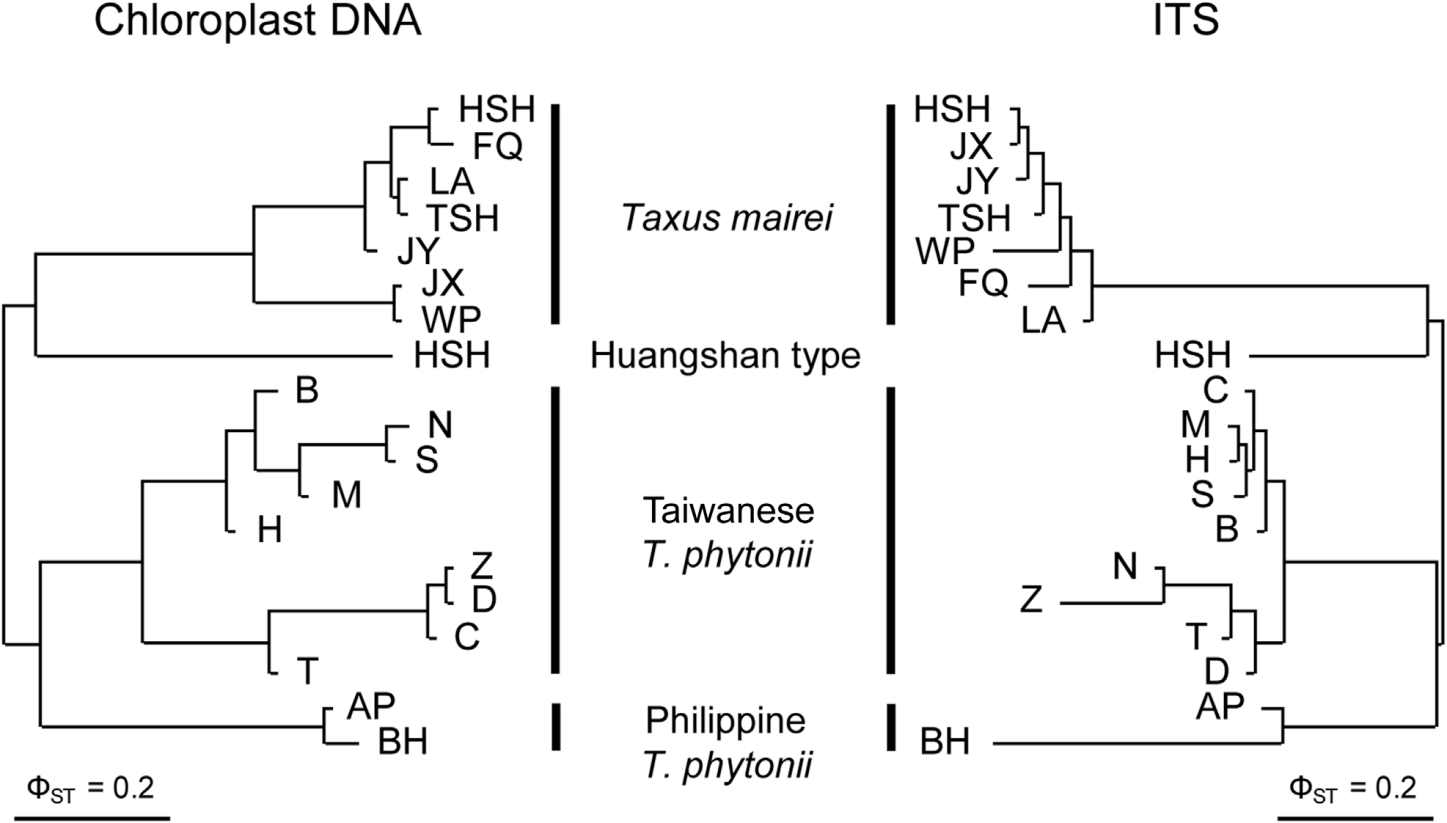
Neighbor-joining clustering trees of Taiwanese *T. phytonii*, Philippine *T. phytonii*, *T. mairei*, and Huangshan-type populations based on chloroplast DNA (left) and nuclear ITS (right) genetic variation. Trees built using pairwise Φ_ST_ between populations.

Constructed with pairwise Φ_ST_ values, the cp- and ITS-based NJ trees consistently grouped the 19 studied populations by taxon (Figure 2), suggesting the four taxa to be genetically well-distinct from one another. Concordant to these findings, cp- and ITS-based AMOVA both assigned much greater fractions of the total genetic variation to inter-taxon differentiation (86.9% and 75.1% for cp- and ITS-based analyses, respectively) than to inter-population differentiation within taxa (5.5% and 3.0% for cp- and ITS-based analyses, respectively) despite significant genetic structure detected at these two hierarchical levels (permutation tests, *p* < 0.001; Table 2). Overall, the above population clustering analyses indicate each of Taiwanese *T. phytonii*, Philippine *T. phytonii*, *T. mairei*, and Huangshan type, representing a genetically distinct unit.

**TABLE 2:** Chloroplast (cp) DNA- and ITS-based analyses of molecular variance (AMOVA) for hierarchical genetic structure among and within Taiwanese *T. phytonii*, Philippine *T. phytonii*, *T. mairei*, and Huangshan type. Fixation indices Φ_CT_ and Φ_SC_ measure genetic divergence among taxa and among populations within taxa, respectively, while Φ_IS_ measures inbreeding extent within populations. *p*-values are obtained through permutation tests (one-tailed tests with 2,000 permutations for each).

Marker	Source of variation	Variation %	Fixation index	*p*-value
CpDNA	Among taxa	86.9	Φ_CT_ = 0.869	<0.001
	Among populations within taxa	5.5	Φ_SC_ = 0.420	<0.001
	Within populations	7.6		
ITS	Among taxa	75.1	Φ_CT_ = 0.751	<0.001
	Among populations within taxa	3.0	Φ_SC_ = 0.119	<0.001
	Among individuals within populations	−1.7	Φ_IS_ = −0.078	0.966
	Within individuals	23.7		

### Haplotype genealogies connecting the East Asian yew taxa

**FIGURE 3:**
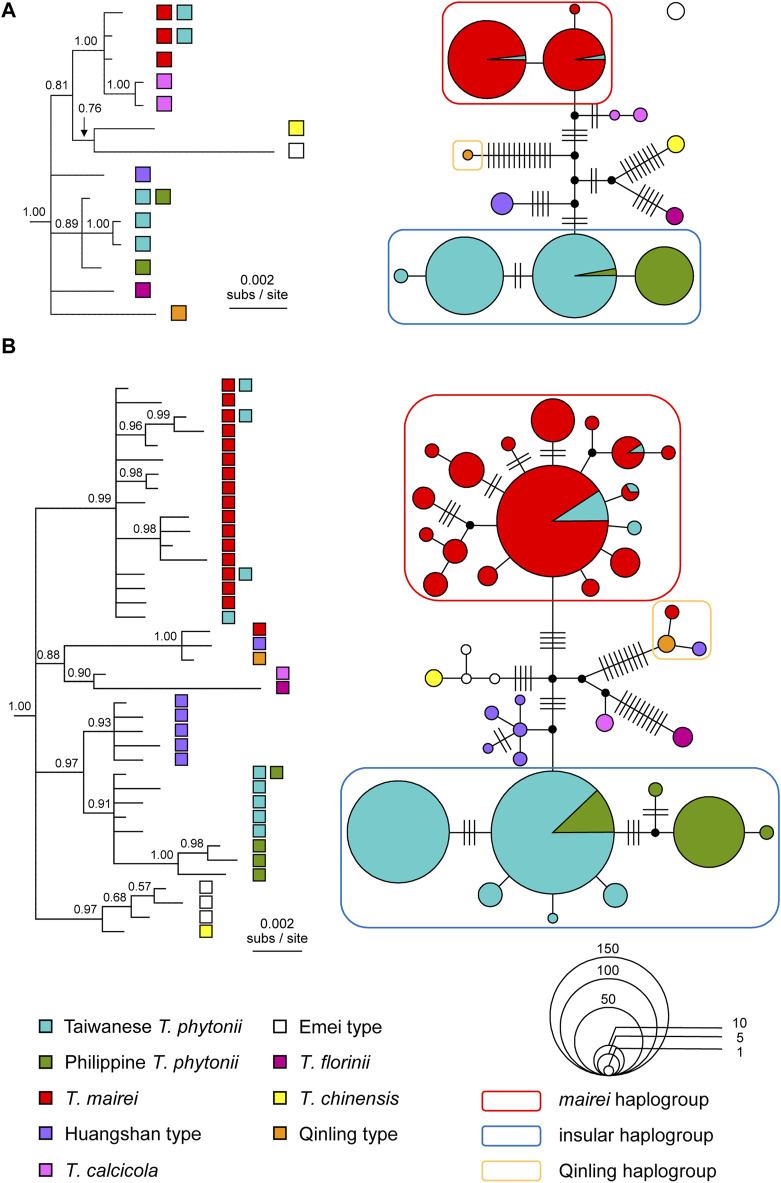
Bayesian consensus trees and statistical parsimony networks reconstructed based on **(A)** chloroplast DNA and **(B)** ITS haplotype sequences from selected East Asian *Taxus* taxa. The trees are rooted with *Pseudotaxus chienii* and *Cephalotaxus manni*. Tree tips are labeled with the source taxa of the haplotypes. Node support values are shown above branches as posterior probabilities. The networks, which are unrooted, have individual haplotypes shown by either filled circles or pie charts, colored according to the source taxa and sized in proportion to the sample sizes. Hatch marks denote mutational steps that separate different haplotypes. It should be noted that the statistical parsimony connection algorithm groups cp haplotypes into two separate networks, consisting of a single haplotype from the *Taxus* Emei type (an empty dot without any connection to all other haplotypes) and all the other haplotypes.

As consistently revealed by the reconstructed haplotype trees and networks, East Asian *Taxus* typically had haplotypes grouping according to taxon identities (Figure 3). The recovered genealogies verified that the two insular yew taxa, Taiwanese *T. phytonii* and Philippine *T. phytonii*, represented each other’s closest relative (Figure 3). Considering together with the fact that Taiwan and the Philippines were constantly isolated from each other by the Luzon Strait, the aforementioned finding implies a colonization history of either an earlier origin of Taiwanese *T. phytonii* which later sent migrants to give rise to Philippine *T. phytonii* or the other way round.

Among identified haplotype groups, one group almost exclusively comprised *T. mairei* sequences but with a small number of sequences from Taiwanese *T. phytonii* (the ‘*mairei* haplogroup’ in Figure 3). There was a second haplotype group which contained all the rest Taiwanese *T. phytonii* sequences plus all Philippine *T. phytonii* sequences (the ‘insular haplogroup’). Within this latter haplogroup, all except one haplotype were exclusive to either Taiwan or the Philippines; the only shared haplotype of these two regions was predominant and widespread in Taiwan, while it was less abundant and restricted to Mt. Apo (AP), Mindanao of the Philippines (Figure 3 and Supplementary Figure S1 of Supplementary Appendix S2). These cases of lineage sharing between *Taxus* taxa, the rarer haplotypes in Taiwan belonging to the *mairei* haplogroup and the rarer haplotype in the Philippines belonging to the insular haplogroup, could result from recent migration events that brought genes from one taxon to another one, more specifically, from *T. mairei* to Taiwanese *T. phytonii* and from Taiwanese *T. phytonii* to Philippine *T. phytonii*. Alternatively, shared lineages could be a legacy of those present in the common ancestor of divergent taxa, namely, incomplete lineage sorting. To distinguish between these alternative explanations, we further applied an IM analysis to *T.* *mairei*, Taiwanese *T. phytonii*, and Philippine *T. phytonii* (see below).

Our ITS-based genealogical reconstructions identified a haplogroup that was remotely related to all the others, comprising a single haplotype from the *Taxus* Qinling type and other two each from one of *T. mairei* and the Huangshan type (the ‘Qinling haplogroup’ in Figure 3B). The latter two haplotypes each represented a one-mutational-step outer edge of the Qinling-type haplogroup (Figure 3B), indicating them to be recent descendants of the Qinling type haplotype. In other words, there were recent introgression events from Qinling type into *T. mairei* and Huangshan type. We excluded the introgressed *T. mairei* haplotype from the subsequent IM analysis which focused on potential migration events among *T. mairei*, Taiwanese *T. phytonii*, and Philippine *T. phytonii*.

### Detections of post-split migrations among *Taxus mairei* and the two insular *T. phytonii* populations

**FIGURE 4:**
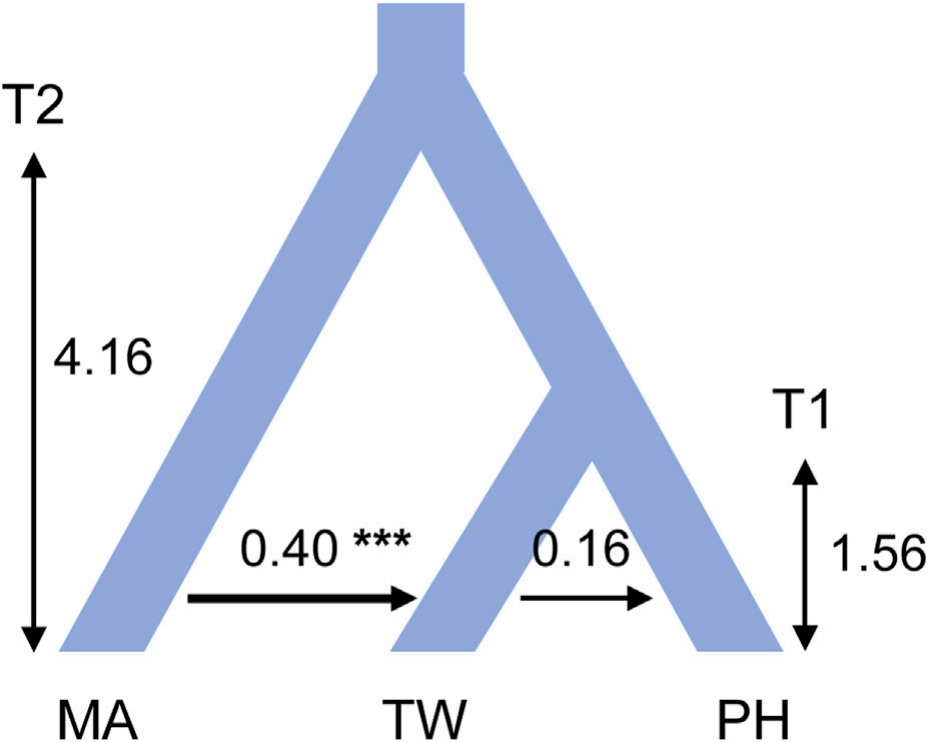
Modal estimates of divergence times and post-split migrations from an isolation with migration analysis that models the divergence process among *Taxus mairei* (MA), Taiwanese *T. phytonii* (TW), and Philippine *T. phytonii* (PH) populations. Sequential divergence times T1 and T2 are in million years. Post-split migrations, with their directions indicated by the arrows, are migrant autosome numbers per generation. The plot only shows post-split migrations that have modal estimates of more than zero (*** denotes *p*-value of <0.001 from a likelihood-ratio test). Demographic parameters described above have their associated 95% highest probability density intervals provided in Table 3.

Consistent estimations were obtained from the three IM replicate runs, which were then combined to obtain a final result. Among the post-split migration parameters modeled, we obtained non-zero modal estimates for *T. mairei* to Taiwanese *T. phytonii* migrations (2N_e_ × M = 0.40 per generation) and Taiwanese *T. phytonii* to Philippine *T. phytonii* (2N_e_ × M = 0.16 per generation; Figure 4 and Table 3), suggesting the presence of migration events. The likelihood ratio tests gave strong statistical support for the *T. mairei* to Taiwanese *T. phytonii* migrations (*p* < 0.001) but not for the Taiwanese *T. phytonii* to Philippine *T. phytonii* migrations (*p* = 0.066; Table 3). We obtained modal estimates for the effective population sizes (N_e_) of *T. mairei*, Taiwanese *T. phytonii*, and Philippine *T. phytonii* as 16,400, 6,900, and 2,700 individuals, respectively (Table 3). We obtained an estimate of the divergence time between Taiwanese *T. phytonii* and Philippine *T. phytonii* accompanied with a very broad confidence interval as 1.56 Mya (95% HPD, 0.006–3.52 Mya); additionally, we obtained a modal estimate of the divergence time of 4.12 Mya (95% HPD, 1.51–4.16 Mya) between *T. mairei* and the common ancestor of the two insular taxa at the set parameter upper bound (Table 3). We emphasize that rather than reflecting an inappropriately set upper bound of the parameter, the reaching of the modal value to the upper bound resulted from an interaction between the priors and the fixed topology of the inter-taxon splits (which imposed a constraint on the sequential divergence times). We demonstrated this by performing an additional IM analysis without inputting data (the ‘prior-only’ analysis). We obtained from this prior-only analysis a modal estimate of the corresponding parameter reaching the set upper bound as in the formal run (Supplementary Figure S2 of Supplementary Appendix S2). In general, the acquired time estimates reflected limited power from a two-locus dataset in resolving the focal parameters with IM models (Hey, 2010).

**TABLE 3:** Isolation with migration (IM) analysis for the divergence process among *T. mairei* (MA), Taiwanese *T. phytonii* (TW), and Philippine *T. phytonii* (PH). Estimates of demographic parameters are shown in consecutive rows as modes, lower 95% highest probability density limits (95HPDIs) and upper 95HPDIs. For post-split migration rates (2N_e_M), values are shown in the fourth row for the likelihood ratio test statistic^a^.

2N_e_M						N_e_			T	
MA→TW	TW→MA	MA→PH	PH→MA	TW→PH	PH→TW	MA	TW	PH	T1	T2
0.40	0	0	0	0.16	0	16.41	6.91	2.72	1.56	4.16
0.13	0	0	0	0	0	8.97	3.09	0.78	6.25E-3	1.51
0.89	0.30	0.21	0.28	0.51	0.51	27.53	13.91	8.28	3.52	4.16
104.26***	0	0	0	2.28	0					

2N_e_M, population migration rate (number of autosomes as migrants per generation); N_e_, effective population size (thousand individual plants); T1 and T2, divergence times (million years ago) between Taiwanese *T. phytonii* and Philippine *T. phytonii* and between *T. mairei* and the common ancestor of the former two taxa, respectively. ***, *p* < 0.001.

^a^ The testing statistic is -2log(Λ), where Λ is the likelihood ratio of the null (zero migration) over the alternative hypothesis (non-zero migration); the *p*-value at α = 0.05 is derived by comparing the testing statistic to a critical value from a 50:50 mixture of χ2(df = 0) and χ2(df = 1) = χ2(df = 1, α = 0.1) = 2.71 (Nielsen and Wakeley, 2001).

### Movements of *Taxus lineages* between Taiwan and the Philippines

**FIGURE 5:**
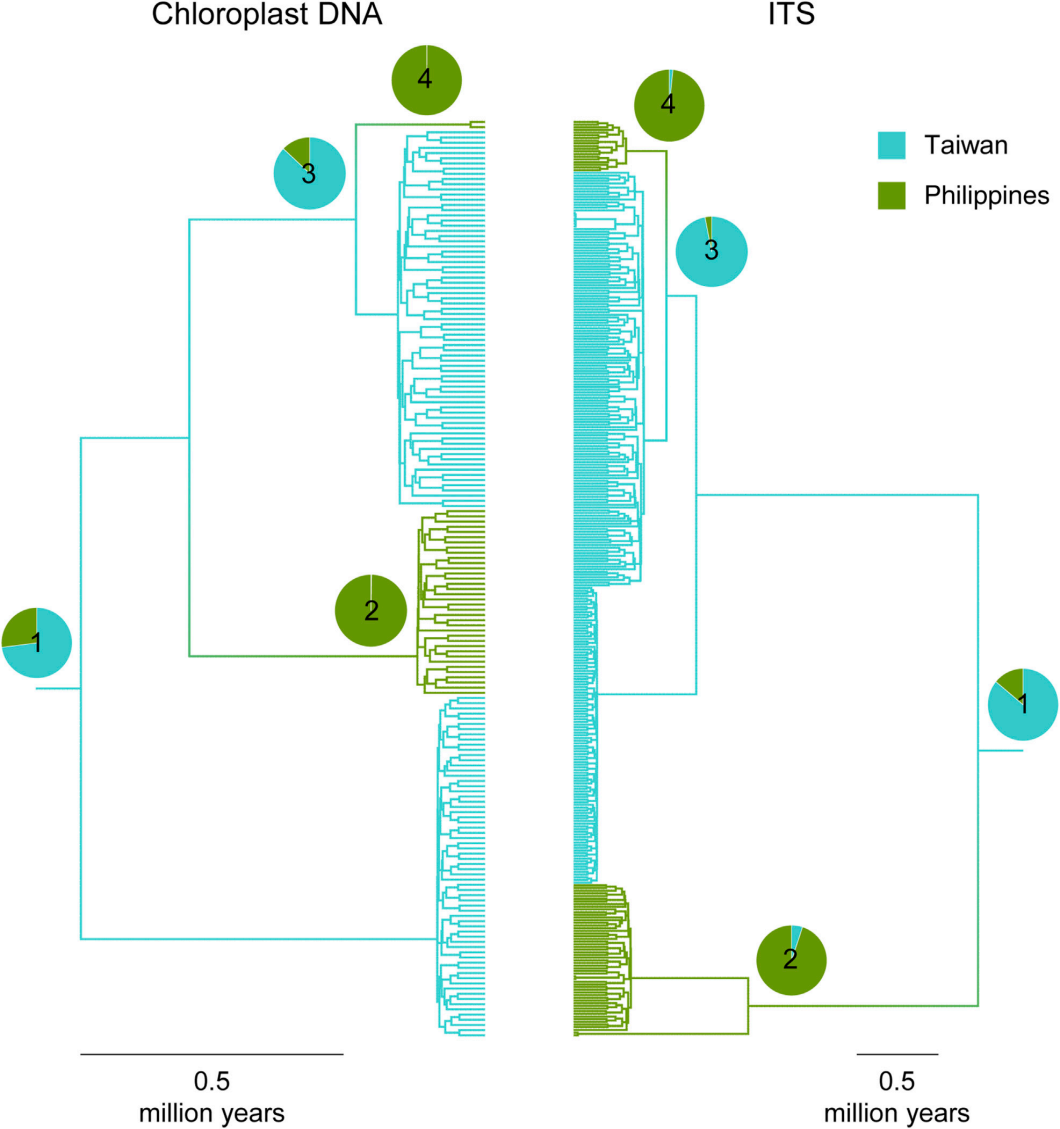
Multi-type tree inferences on ancestry locations (either Taiwan or Philippines) of Taiwanese *T. phytonii* and Philippine *T. phytonii*. Plots show maximum clade credibility coalescent trees, with individual lineages colored according to the most likely locations inferred. For selected tree nodes (#1–4), the full posterior distributions regarding their locations are presented by pie charts. Time estimates associated with these selected tree nodes are provided in Supplementary Table S6 of Supplementary Appendix S2.

Both cp- and ITS-based MTT analyses inferred that there were most likely two incursion events from Taiwan to the Philippines but zero from the Philippines to Taiwan (Supplementary Figure S3 of Supplementary Appendix S2). Visualized on the gene trees (Figure 5), the more ancient Taiwan-to-Philippine incursion event resulted in lineages currently distributed in both Philippine sites BH and AP (cp haplotype #8 and ITS haplotypes #28–30 in Supplementary Figure S1). This incursion event, presumably corresponding to the origin of Philippine *T. phytonii*, was followed by a more recent one in the same direction that contributed to the rarer Philippine *T. phytonii* lineage found in AP (cp haplotype #5 and ITS haplotype #23 in Supplementary Figure S1).

Regarding the MTT-recovered evolutionary history, we provided date estimates for some time points, described as follows. First, the most recent common ancestor (MRCA) of all Taiwanese *T. phytonii* and Philippine *T. phytonii* lineages (node #1 in Figure 5) was dated to 0.77 and 2.49 Mya based on cpDNA and ITS, respectively (mean estimates, with associated 95% confidence sets provided in Supplementary Table S6 of Supplementary Appendix S2). This provided a lower limit when Taiwanese *T. phytonii* had already existed. Second, the MRCA of the more-ancient lineages of Philippine *T. phytonii* (node #2 in Figure 5) was dated to 0.13 and 1.08 Mya based on cpDNA and ITS, respectively. Likewise, Philippine *T. phytonii* had originated before this date. The third and the fourth time points, nodes #3 and #4 in Figure 4, respectively, bracketed the duration when Taiwanese *T. phytonii* secondarily emerged in the Philippines; the arrival of AP there was dated to 0.03–0.25 and 0.32–0.57 Mya based on cpDNA and ITS, respectively.

## Discussion

### Origin and colonization routes of insular *T. phytonii*

In this study, population-based pairwise NJ clustering trees from both cpDNA and nuclear markers consistently indicated Taiwanese *T. phytonii* and Philippine *T. phytonii*, each representing a genetically distinct unit which was also distinct from the other Chinese mainland-related *Taxus* populations, *T. mairei*, and the Huangshan type (Figure 2; Table 2). This relationship was not completely conclusive in previous phylogeographic studies (Gao et al., 2007; Zhang et al., 2009; Möller et al., 2020) or Global/Eurasian yew barcoding analysis (Liu et al., 2011; Liu et al., 2018) due to insufficient or no sampling of both the two insular *T. phytonii* populations. Furthermore, we dissected the historical genetic relationships between Taiwanese *T. phytonii* and Philippine *T. phytonii*, with cp- and ITS-based haplotype genealogies based on samples from nine Taiwanese populations (total n = 141; Table 1) and two Philippine populations (total n = 38). The result revealed a sister relationship between Taiwanese *T. phytonii* and Philippine *T. phytonii* to the exclusion of other East Asian *Taxus* (Figure 3). This was followed by a conclusion from the cp- and ITS-based MTT analyses which inferred the genes’ most recent common ancestors (MRCAs) to be more likely located in Taiwan (Figure 5) and thus supported that Philippine *T. phytonii* was derived from Taiwanese *T. phytonii*.

As to the origin of *T. phytonii*, Möller et al. (2020) suggested a southward diversification wave of *Taxus* from Central China, with one of several lineages crossing the Taiwan Strait at ∼3.5 Mya to form *T. phytonii*. This time estimate may be quite reliable because the reconstructed dated phylogeny used 13 chloroplast genes and two nuclear markers and included all 16 extant *Taxus* taxa lineages of the world. However, the sequential establishments of *T. phytonii* populations, implied by the above suggestion to be first in Taiwan then in the Philippines, had not been formally tested. Empowered by comprehensive sampling over the two insular regions, our results first time confirmed that Taiwanese *T. phytonii* sent migrants to colonize the Philippines as a part of the southward *Taxus* range expansion out of Central China, one of this taxon’s diversity core regions (Möller et al., 2020).

### Introgression from *Taxus mairei* to Taiwanese *T. phytonii* and Taiwan-to-Philippines incursions

With DNA sequences from other Chinese mainland *Taxus* analyzed together, the reconstructed cp- and ITS-based haplotype genealogies revealed *T. mairei*-related haplotypes in Taiwan (Figure 3), suggesting recent migrations from *T. mairei* to Taiwanese *T. phytonii* or retentions of ancestral polymorphism. Using the likelihood ratio test adjoined to the IM analysis, we then gained strong support for the presence of recent migrations (MA- > TW migration rate = 0.40, 95% HPD: 0.13–0.89, Table 3), which due to their occurrence between divergent taxa (visualized in Figure 4), were commonly called post-split migrations or introgression.

Similarly, both cp- and ITS-based haplotype genealogies and the IM analysis suggested recent secondary migrations from Taiwanese *T. phytonii* to Philippine *T. phytonii* (TW- > PH migration rate = 0.16, 95% HPD: 0–0.51, Table 3 and Figures 3, 4). Moreover, the cp- and ITS-based MTT analyses further inferred the occurrence of a second-time lineage incursion event from Taiwan to the Philippines (Figure 5 and Supplementary Figure S3 of Supplementary Appendix S2), which occurred within the last 0.6 million years (bracketed by MTT tree nodes #3 and #4). This latter incursion event resulted in a cp and an ITS haplotype that were commonly found in Taiwan and Mindanao but not in Luzon (Figure 3; Supplementary Figure S1 of Supplementary Appendix S2), implying recent long-distance dispersal of Taiwanese *T. phytonii* to Philippine *T. phytonii.*

With seeds being able to be primarily transported by frugivorous birds endozoochorously (Martinez et al., 2008; Li et al., 2014; Li et al., 2015; Lavabre et al., 2016) and possibly secondarily transported (diploendozoochory) by raptors which prey on and potentially undertake even farther-reaching flight than frugivorous birds (Hamalainen et al., 2017), *Taxus* may have an extended capacity of overseas dispersal. We speculate the initial establishment of Philippine *Taxus* by transportation of seeds of Taiwanese *Taxus* at least across the full length of the Luzon Strait (>250 km) to reach a suitable habitat such as those surviving Philippine *T. phytonii* individuals at the summit of Mt. Banahaw (2,158 asl) in the Luzon island because no other high mountains left in between. Until our study, commuting of non-volant, terrestrial taxa between Taiwan and main islands of the Philippines was only genetically evidenced in the herb *Euphrasia*, which were also inferred to colonize Luzon from Taiwan (Wu et al., 2009). Here, the small seeds (1–1.5 mm long, Wu et al., 2009) are considered to be transported by birds epizoochorously. It remains to be clarified how *Taxus* seeds were secondarily transported from Taiwan to Luzon, and to Mindanao.

Many bird species use Taiwan as a stopover during their autumn southward migrations to the wintering sites in the Philippines and other places (Shiu and Lee, 2003; Yong et al., 2015). These include thrushes of the genus *Turdus* whose European congeners are the main disperser of *Taxus* seeds (Martinez et al., 2008; Lavabre et al., 2016). A peak of thrush arrival has been shown in Taiwan at November–December (Chen et al., 2020), matching the period when Taiwanese yews’ fruits (the cup-like arils surrounding the seeds) turn red (Wang and Chien, 2016). Assuming a similar ripening time of Philippine yews’ fruits, this time would be discrepant from that when the birds start to return northerly at April–May (Billerman et al., 2022). Therefore, the prevalent bird migration patterns in the East Asia could be responsible for the observed unidirectional *Taxus* introgression from Taiwan to the Philippines. Alternatively, the observed MA- > TW and TW- > PH migrations, but not the other way round, could be simply caused by unequal population sizes of these two insular yew populations; between pairs of populations with contrasting sizes, migrations in the two directions would be imbalanced because larger populations send out more migrants than smaller populations do. In line with this second explanation, our IM analysis inferred the largest population in the mainland (*T. mairei*), followed by a smaller one in the subtropical island (Taiwanese *T. phytonii*) and the smallest one in the remote tropical islands where suitable habitats are rare and isolated (Philippine *T. phytonii*) (Table 3).

### Divergence time estimation of insular *T. phytonii*

The MTT gave uniformly larger age estimates for corresponding tree nodes in the ITS tree than in the cp tree (Supplementary Figure S6 of Supplementary Appendix S2). We attribute this in part caused by the four-fold effective population size difference between the biparentally inherited autosomal maker (ITS) and the uniparentally inherited plastid marker (cpDNA); according to the coalescent theory (Kingman, 1982), a larger effective population size renders a longer time to reach the common ancestor by coalescence. Since the node ages present timing when their descendant lineages had already existed (and so the taxon bearing these lineages), we use the ITS-derived ages as the lower limits of taxon origin dates.

We, thus, infer the origin of Taiwanese *T. phytonii* predating 2.49 Mya (the mean age for the MTT tree node #1; visualized in Figure 5); the confidence interval associated with this node age (Supplementary Figure S6 of Supplementary Appendix S2) overlapped with 3.5 Mya from Möller et al. (2020)’s estimation for *T. phytonii*’s origin. The estimated origin of Taiwanese *T. phytonii* at 2.49–3.5 Mya implies this taxon to be among the first colonizers that migrated to Taiwan, along with other conifers such as *Taiwania* (1.9–2.5 Mya) and *Chamaecyparis* (1.3–2.9 Mya) (Wang et al., 2003; Chou et al., 2011).

Frequent land bridges were emerged in connecting Chinese mainland and Taiwan during glaciation maximum in Pliocene and Pleistocene due to the shallow sea level of the Taiwan Strait (Ota, 1998). Specifically, the proximity of the island to the continent (<200 km) with a shallow continental shelf in between (<100 m in depth) provided chances to immigrations from the continent over land bridges during any glacial periods when the sea level dropped (Voris, 2000). Repeated isolation and reconnection during glacial–interglacial cycles resulted in the co-occurrence of divergent lineages within the Taiwan Island that reflected separate immigration events. Indeed, a range of Taiwanese animals and plants showed such phylogeographic patterns (Yu, 1995; Tzeng et al., 2006; Chiang et al., 2012; Kuo et al., 2015), including *Taxus* documented here. On the other hand, *Taxus*, given a hypothetically great capacity of bird-mediated long-distance dispersal as described above and with such capacity suggested by the Taiwan-to-Philippines colonization (see discussion below), may not need the emergences of land bridges for colonization across the Taiwan Strait. It should be noted that the glacial–interglacial cycles were dominated by a periodicity of only 41 thousand years before mid-Pleistocene (∼0.8 Mya) (Tziperman and Gildor, 2003). The uncertainty associated with the estimated origin time of Taiwanese *T. phytonii* (2.49–3.5 Mya) is too large with respect to such a short periodicity to clarify whether colonization into Taiwan occurred during glacial or interglacial periods.

We infer an origin of Philippine *T. phytonii* earlier than 1.08 Mya (the mean age for the MTT tree node #2). Philippine islands were volcanic, oceanic, and surrounded by sea that is too deep to form land bridges even when the sea level dropped during glacial maxima (Voris, 2000). The unusually shared floristic elements between Taiwan and the Philippines suggest that the haplotypes of Philippine *T. phytonii* could be either directly decent from continental Asia *Taxus*, which is unlikely given the remote distance of Philippine islands which were never connected to continental Asia, or more likely, colonized from Taiwanese *T. phytonii*, as revealed from our results (Figures 3, 5).

### Repeated occurrences of higher introgression levels at ITS than at cpDNA and the possible explanations

In addition to aforementioned findings, we noticed systematic differences between cpDNA and ITS in their introgression levels across *Taxus* taxa, suggesting common drivers underlying such between-marker variations. Specifically, the introgression from *T. mairei* to Taiwanese *T. phytonii*, revealed by the occurrence of *T. mairei*-related haplotypes in Taiwan, showed a higher level at ITS than at cpDNA: 16/282 (5.7%) ITS and 2/141 (1.4%) cp sequences represent introgressed sequences. Similarly, 19/76 (25.0%) secondarily incurred (=introgressed) ITS sequences from Taiwanese *Taxus* to Philippine *Taxus* were proportionally more abundant than the 2/38 (5.3%) introgressed cp sequences in the same direction. Still, the third example was made of introgression from the *Taxus* Qinling type to each of the *T. mairei* and *Taxus* Huangshan type, with each case showing ITS introgression accompanied with no cpDNA introgression (Figure 3). For *Taxus*, a dioecious plant with paternal cpDNA inheritance, such a pattern is incompatible with scenarios where introgression is solely or mainly realized through pollen dispersal; this is because any individual rising as a seed fertilized by a flowed-in pollen would have its whole genetic composition at a cp locus introgressed but only half that at a nuclear locus introgressed, causing an instantaneous introgression rate that is double at the former locus compared to that at the latter locus. Rather, a combination of the following two mechanisms could explain the observed higher nuclear versus cp introgression levels for *Taxus*: (1) introgression is solely or mainly realized through seed dispersal and (2) the female-to-male sex ratio in the individuals rising as incurred seeds is higher than that in those produced by the local taxon. Below, we elaborate the mechanisms in further detail.

Unlike pollen dispersal-mediated introgression, which involves only male trees of the donor taxon, seed dispersal-mediated introgression involves both male and female trees of the donor taxon, occurring through hybridization when these trees (rising from incurred seeds) become sexually mature. In brief, since female *Taxus* cannot pass cpDNA down to the next generation, a higher proportion of exotic female trees than local female trees in the recipient taxon would give disadvantage to the pass-down of exotic cp genes but not of exotic nuclear genes in relative to local genes of respective types, hence a higher introgression level at a nuclear than at a cp locus. In ‘Introgression levels at chloroplast and biparental nuclear loci for *Taxus*’ (Supplementary Appendix S3 and adjoint Supplementary Figure S4; Supplementary Figure S5), we raise equations for the dynamics of cp and nuclear introgression levels under circumstances where, respectively, pollen dispersal alone and seed dispersal alone are in charged for the introgression; these equations take into account inheritances of introgressed genes over generations. We demonstrate with these equations that the two mechanisms proposed above together are capable of producing the observed higher introgression at ITS than at cpDNA.

Although considered a wind-pollinated plant (Thomas and Polwart, 2003), *Taxus* might have a reduced propensity for long-distance pollen transportation, given that individual yew trees typically reside under the canopy of the forest where wind velocity and turbulence are low (Allison, 1990). In contrast, *Taxus* may have a good propensity for long-distance seed transportation, as discussed earlier. Following seed transportation, sex ratio-adjusting mechanisms (SRAMs) that differentially act on exotic and local seeds or on their progeny are supposed to take place, causing a higher female-to-male ratio in the exotic elements than in the local elements and, in turn, leading to our observation of higher introgression at ITS than at cpDNA. Such SRAMs could act on flowed-in seeds or on individual plants rising from these seeds before they are sexually matured (Shelton, 2010a; Shelton, 2010b), resulting in proportionally more exotic female trees to survive than local female trees. Alternatively, SRAMs could act in producing more female-determining pollen via a meiotic drive (Taylor and Ingvarsson, 2003) or in increasing siring success of female-determining pollen (Stehlik and Barrett, 2006), hence a female-biased sex ratio result. Nevertheless, nothing has been known about the performance of these SRAMs in the context of inter-taxon hybridization and whether they could differentially perform in creating a more extreme female-biased ratio in the hybrids than in the purebreds. Finally, theories (reviewed in Dufresnes et al., 2016) and empirical evidence (Ouyang et al., 2010; Pucholt et al., 2017) have been raised or gathered for processes responsible for higher hybrid genetic incompatibility in male than in female trees, which leads to a female-biased sex ratio in hybrids. Such SRAMs could have acted on *Taxus* hybrids, leaving proportionally more female trees in the hybrids than in the purebreds of the focal taxon.

### Conservation of insular *Taxus*

Altogether, we exemplified with yew trees that Taiwan, given its geographic position and topography, served as a stepping stone connecting its surrounding landmasses. Taiwan is a continental island which is close to the Chinese mainland (Taiwan Strait <200 km wide), facilitating many plants to migrate into Taiwan, possibly via land bridges emerging during glacial maxima (Wang et al., 2004; Chou et al., 2011). In addition, Taiwan is characterized by harboring mountains of >1,000 m a.s.l. for approximately one-third of its land area, which renders the island a potent refugium to temperate-originated organisms (Cheng et al., 2005). This, in turn, gave opportunities to new taxon derivations in the tropical mountains once long-distance colonization was feasible, a scenario demonstrated here by *Taxus* and *Euphrasia*.

Both Taiwanese and Philippine *Taxus* populations are located in biodiversity hotspots for conservation priorities (Myers et al., 2000). As a genetically well-distinct southward migration lineage, Taiwanese *Taxus* and Philippine *Taxus* (*T. phytonii*) may have populations particularly vulnerable compared to continental relatives due to limited highly suitable habitats on these islands. Indeed, our IM estimated an N_e_ value of Taiwanese *Taxus* ∼2.4× (∼6× in case of Philippine *Taxus*) smaller than that of *T. mairei* from SE mainland China (Table 3). This is evident as we can only manage to collect Philippine *Taxus* samples at mountain summits in Luzon and Mindanao each with less than 30 individuals were known to survive. As a result, these leading-edge subtropical and tropical populations may suffer genetic drifting and are likely to be at risk from climate change. Moreover, these Taiwanese and Philippine genotypes are unique lineages different from all other East Asian *Taxus* lineages. These populations are also the southward and the rare oceanic extensions of *Taxus* distribution, which may preserve genetic alleles for resisting heat stress. This could be an important adaptive trait for *Taxus* survival for global warning. Therefore, it is critical to include *T. phytonii* into global yew conservation management, as revealed from this study, given previously no suggestion was provided. Future studies that are powered with genome-wide variation data (e.g., AFLP, RADseq, and phylogenomics) regarding genetic variability levels within local populations will render broadly consensus evidence and be warranted for conservation of insular *Taxus*.

## Data Availability

The original contributions presented in the study are included in the article/Supplementary Material; further inquiries can be directed to the corresponding author.
