# Supplementary material for: A leading-edge scenario in the phylogeography and evolutionary history of East Asian insular Taxus in Taiwan and the Philippines
Source: Front Genet. 2024 May 2;15:1372309. doi: 10.3389/fgene.2024.1372309 (PMC11096487; doi:10.3389/fgene.2024.1372309)
Supplement: Supplementary file 1 [file DataSheet1.DOCX]

**A leading edge scenario in the phylogeography and evolutionary history of East Asian insular *Taxus* in Taiwan and the Philippines**

Hao-Chih Kuo^1†^, Travis Schoneman^1†^, Lian-Ming Gao^2^, William Sm. Gruezo^3^, Victor B. Amoroso^4^, Yang Yang^5^, Kuo-Cheng Yang^6^, Ching-Te Chien^7^, Michael Möller^8^, and Chun-Neng Wang^1,9,*^

^1^Institute of Ecology and Evolutionary Biology, National Taiwan University, Taipei 10617, Taiwan; ^2^Key Laboratory for Plant Diversity and Biogeography of East Asia, Kunming Institute of Botany, Chinese Academy of Sciences, Yunnan 650201, China; ^3^Plant Biology Division, Institute of Biological Sciences, College of Arts and Sciences, University of the Philippines at Los Baños, College, Laguna, Philippines; ^4^Center for Biodiversity Research and Extension in Mindanao (CEBREM) and Central Mindanao University, Mindanao, the Philippines; ^5^Tainan District Agricultural Research and Extension Station, Ministry of Agriculture, Taiwan; ^6^General Education Center, Providence University, Taichung, Taiwan; ^7^Botanical Garden Division, Taiwan Forestry Research Institute, Taipei 10066, Taiwan; ^8^Royal Botanic Garden Edinburgh, EH3 5LR, Scotland, UK; ^9^Department of Life Science, National Taiwan University, Taipei 10617, Taiwan.

^†^These authors contribute equally to this manuscript

^*^Corresponding author

**Supplementary materials**

APPENDIX S1

**Marker amplifications, sequencing and ITS haplotype reconstructions**

The same PCR protocol was used for amplifications of all markers. The 30 μL reaction mix contained 50-100 ng template DNA, 3 μL of 10× *Taq* buffer (MD-BIO Inc., Taiwan), 2.4 μL of 2.5 mM dNTP mixture (Genemark, Taiwan), 1.5 μL each of 10 mM forward and reverse primers, and 0.24 μL of *Taq* DNA polymerase (MD-BIO Inc.). Primer pairs for corresponding markers were listed in Table S1. Performed with an ABI 2720 Thermal Cycler (Applied Biosystems, USA), the following thermal profile was used: 94°C 3 min; 35 cycles of 94°C 45 s, 55°C 45 s and 72°C 1.5 min; 72°C 7 min. PCR products were checked with 1% TBE agarose gel, purified with a Viogene Gel/PCR DNA Isolation System kit (Viogene, Taiwan) and sequenced on an ABI 3730 XL DNA Analyzer (Applied Biosystems).

Approaches as follows were adopted in resolving the two ITS haplotypes for individual plants each showing at this marker as a heterozygote. First, Flot *et al.*’s (2006) approach was applied to each that showed indel polymorphism. Briefly, when read in one direction, each such individual would be characterised by having double peaks on the chromatogram only downstream the indel; complementary information from the forward and reverse chromatograms can thus be used to resolve the part showing superposed nucleotide signatures. A number of internal primers (listed in Table S1) were used for obtaining clear forward and reverse chromatograms that flanked specific indels, and the two haplotypes per individual were resolved with the aid of the online program Indelligent (Dmitriev & Rakitov 2008).

Second, among heterozygous individuals that showed only single-nucleotide polymorphisms, the Bayesian algorithm implemented in PHASE 2.1 (Stephens & Donnelly 2003; Stephens *et al.* 2001) was used for those each containing two or more polymorphic sites (the two haplotypes for each of those showing only one single-nucleotide polymorphic site were directly obtained). With the PHASE input prepared in SeqPHASE (Flot 2010), we performed ten replicate runs of Markov chain Monte Carlo (MCMC), each run for 15,000 iterations (10 iterations per sample) under the model allowing intragenic recombination. Already known haplotypes, including those inferred from indel polymorphisms as described above, were included to facilitate phasing. For each individual showing convergent results across replicate runs, its inferred pair of haplotypes was accepted when such a pair received an estimated posterior probability (PP) of >0.9.

Finally, for heterozygous individuals unable to be resolved using either of the former two approaches, such samples were resolved experimentally via TA-cloning. To this end, PCR was performed as described above except that a Phusion High-Fidelity *Taq* (NEB, USA) was used in replacement of the normal *Taq*, so to reduce PCR-induced nucleotide changes; the initial denaturation of such PCR was at 98°C following the manufacturer’s instruction. The PCR products were ligated to the pGEM-T Easy Vectors (Promega, USA) and were together transformed into the HIT^TM^-DH5α competent cells (RBC Bioscience, Taiwan) by heat shock. Transformants were grown overnight in Luria-Bertani (LB) containing 50 ng/μL ampicillin, and successfully transformed colonies were initially selected via white-blue screening followed by confirmation with amplifications and gel electrophoresis. The selected colonies were further grown in LB with ampicillin for 10-12 hours, from which the plasmid DNA was then isolated using a Viogene Plasmid DNA Extraction System (Viogene, Taiwan) according to the manufacturer’s protocol. For each individual, we sequenced 8–12 clones to confirm its two ITS haplotypes. With thus experimentally resolved haplotypes included as references, additional ten PHASE runs were performed, so to confirm that every *Taxus* individual had its inferred pair of haplotypes supported with a PP of >0.9.

| **Table S1** Primers used in this study, with internal primers newly designed for aiding resolving ITS heterozygotes marked by star symbols. | | | |
| --- | --- | --- | --- |
| Marker | Primer sequence (5’ to 3’) | Direction | Source |
| *trn*L-F | *trn*c: CGAAATCGGTAGACGCTACG | Forward | Taberlet *et al.* (1991) |
|  | *trn*f: ATTTGAACTGGTGACACGAG | Reverse | Taberlet *et al.* (1991) |
| *trn*H-*psb*A | *trn*H: CGCGCATGGTGGATTCACAATCC | Forward | Tate and Simpson (2003) |
|  | *psb*A: GTTATGCATGAACGTAATGCTC | Reverse | Sang *et al.* (1997) |
| *pet*G-*trn*P | *pet*G: GGTCTAATTCCTATAACTTTGGC | Forward | Hwang *et al.* (2000) |
|  | *trn*P: GGGATGTGGCGCAGCTTGG | Reverse | Hwang *et al.* (2000) |
| *atp*I-H | *atp*I: TATTTACAAGYGGTATTCAAGCT | Forward | Shaw *et al.* (2007) |
|  | *atp*H: CCAAYCCAGCAGCAATAAC | Reverse | Shaw *et al.* (2007) |
| ITS | ITS-Leu: GTCCACTGAACCTTATCATTTAG | Forward | Baum *et al.* (1998) |
|  | ITS4: TCCTTCCGCTTATTGATATGC | Reverse | White *et al.* (1990) |
|  | ITS-i1*: GCACGAAATAATCTGTACGGC | Reverse | This study |
|  | ITS-i2*: GTCTTCGCAAGTTGTTCTCC | Forward | This study |
|  | ITS-i3*: CTGCAACCAGAGGCGATG | Reverse | This study |
|  | ITS-i4*: GGAGTTCGGACTGGAGTTC | Forward | This study |
|  | ITS-i5*: CAAATGTTCAACGGTCTGC | Reverse | This study |
|  | ITS-i6*: CCGTACAGATTATTTCGTGC | Forward | This study |

| **Table S2** GenBank chloroplast genomes that are used in the genealogical analysis of this study. | | |
| --- | --- | --- |
| Taxon | Voucher | Accession |
| ***Taxus*^a^** |  |  |
| *T. calcicola* | GLM164262 | MH390451 |
|  | GLM123951 | MH390461 |
|  | GLM123950 | MH390489 |
| *T. chinensis* | TCP0102 | MH390476 |
|  | TCP0202 | MH390478 |
|  | TCP0303 | MH390442 |
| *T. florinii* | TFP0302 | MH390487 |
|  | TFP0402 | MH390463 |
|  | ML04 | MH390473 |
| Emei type | TEP0101 | MH390456 |
|  | TEP0202 | MH390472 |
|  | TEP0303 | MH390475 |
| Qinling type | QL1 | MH390444 |
|  |  |  |
| **Outgroup** |  |  |
| *Pseudotaxus chienii*^b^ | PCHIE20171209 | MH023407 |
| *Cephalotaxus manni*^c^ | Y. Ji and X. Gong 003 | MT555084 |
| ^a^ Fu et al. (2019); ^b^ Wang et al. (2019); Ji et al. (2021) | | |

| **Table S3** GenBank ITS sequences from Liu et al. (2011) that are used in the genealogical analysis of this study. | | | |
| --- | --- | --- | --- |
| Taxon | Longitude/Latitude | Voucher | Accession |
| ***Taxus*^a^** |  |  |  |
| *T. calcicola* | 105° 35'E/25° 01'N | GLM-06001 | HM590953 |
|  | 104° 46'E/23° 23'N | GLM-2357 | HM590954 |
|  | 104° 38'E/20° 53'N | NVSDE82 | HM590955 |
| *T. chinensis* | 104° 13'E/32° 39'N | LJ-05-999 | HM590947 |
|  | 107° 15'E/32° 48'N | MMO 05-597 | HM590948 |
|  | 110° 05'E/31° 27'N | MMO 05-507 | HM590949 |
| *T. florinii* | 100° 08'E/27° 01'N | GM-2441 | HM590943 |
|  | 099° 10'E/28° 16'N | GM-24171 | HM590944 |
|  | 100° 47'E/27° 36'N | GM-24201 | HM590945 |
|  | 101° 11'E/28° 04'N | GF-040911-29 | HM590946 |
| Emei type | 103° 03'E/28° 43'N | GLM-07704 | HM590950 |
|  | 103° 03'E/28° 43'N | Psh08 | HM590951 |
|  | 103° 15'E/27° 13'N | 03-0606-8 | HM590952 |
| Qinling type | 107° 04'E/33° 51'N | MMO 05-694 | HM590961 |
|  | 111° 14'E/33° 48'N | LJ-05-1024 | HM590962 |
|  | 113° 19'E/35° 45'N | LJ-05-1062 | HM590963 |
|  |  |  |  |
| **Outgroup** |  |  |  |
| *Pseudotaxus chienii*^b^ | - | Ps3 | MT735121 |
| *Cephalotaxus manni*^c^ | - | C111 | JF976108 |
| ^a^ Liu *et al.* (2011); ^b^ Möller *et al.* (2020); China Plant BOL Group *et al.* (2011) | | | |

**Settings for demographic priors**

Demographic parameters of an isolation with migration (IM, Hey 2010; Hey & Nielsen 2007) model include rescaled post-split migration rates (*m*; *m* = M/u where M is the per generation proportion of the population as the migrants, and u is the geometric mean of the locus-wide per generation mutation rates), composite parameters for effective population sizes (*q*; *q* = 4N_e_×u for a population of a diploid organism with N_e_ individuals) and composite parameters for inter-taxon divergence times (*t*; *t* = T×u where T is the divergence time as the number of generations). Truncated uniform priors are implemented in the program IMa2 (Hey 2010; Hey & Nielsen 2007) for all above parameters. We consulted Hey (2011) to set upper bounds of truncated uniform priors, followed by adjustments according to results from exploratory IM runs. Specifically, Hey (2011) suggested upper bounds as 2/*q_max_*, 5×*q_max_* and 2×*q_max_* for *m*, *q* and *t*, respectively, where *q_max_* was the maximum *q* value among those of the focal taxa derived based on autosomal markers; in our case, we derived *q_max_* based on ITS. Using θ_W_ as an estimator of *q*, we obtained *q_max_* = 2.88 per locus (Table S4). Accordingly, we set the upper bound of *t* to 6, slightly higher than 2×*q_max_* (= 5.76). We set the upper bounds of *m* and *q* to 1.5 (higher than 2/*q_max_* = 0.69) and 9 (lower than 5×*q_max_* = 14.40), respectively, after inspections on exploratory run results.

Using haploid organisms as a modeling template, the MultiTypeTree (MTT, Vaughan *et al.* 2014) model incorporates a N_e_×g parameter for each of the focal population, with N_e_ and g representing the effective population size and the generation time (in the same time unit as that used for the tree calibration), respectively. When applied to *Taxus* trees, which have diploid individuals of separate sexes and have cpDNA inherited paternally (Anderson & Owens 1999), N_e_ values of the cp- and ITS-based MTT models refer to the numbers of genes of corresponding markers in a population; therefore, for any specific *Taxus* taxon, N_e_ is four times larger in the ITS-based model than in the cp-based one. In both MTT analyses of this study, we specified truncated uniform priors for N_e_×g parameters, each of which had an upper bound set as 1. This upper bound was selected as an arbitrary value that was much larger than the maximum N_e_×g value among those of the two focal *Taxus* taxa (Taiwan *T. phytonii* and Philippine *T. phytonii*) derived based on the ITS dataset. More specifically, we estimated N_e_×g with the formula N_e_×g = θ/2u, where u was the clock rate, and θ = 2N_e_×u×g for ITS (u×g was in a unit of substitutions per nucleotide site per generation). We obtained a maximum θ value of the two focal taxa as 0.00187 per nucleotide site (Table S4). Calculated with this θ value as well as with the ITS mutation rate as 0.011 substitutions per nucleotide site per 10 million years (see ‘Clock rates of chloroplast DNA and ITS for *Taxus*’), we then obtained a maximum N_e_×g value as 0.085. This N_e_×g value was much smaller than our set upper bound for the truncated uniform prior (= 1).

The MTT model further incorporates the migration rate parameter as *m*i_🡪_j = Mi_🡪_j×N_e_i/N_e_j, where Mi_🡪_j is the accumulated proportion of population j as the migrants from population i in one time unit (= 10 million years in our case), and N_e_i and N_e_j are effective population sizes of populations i and j, respectively. For each such *m*i_🡪_j parameter in either cp- or ITS-based analysis, we specified a lognormal prior with a log(mean) = 1 and log(standard deviation) = 4, which covered a broad range of potential *m*i_🡪_j values. Specifically, this prior setting corresponded to a proportion of migrants per generation ranging from exp(-4)×25/10,000,000 = 4.57E-8 to exp(4)×25/10,000,000 = 1.36E-4 when N_e_i = N_e_j, assuming a generation time of *Taxus* as 25 years (Wang *et al.* 2006).

| **Table S4** ITS-derived Watterson’s (1975) θ_W_ values for *Taxus mairei* (MA), Taiwan *T. phytonii* (TW) and Philippine *T. phytonii* (PH). | | | |
| --- | --- | --- | --- |
|  | MA^a^ | TW | PH |
| per nucleotide site | 0.00251 | 0.00187 | 0.00125 |
| per locus | 2.88 | 2.09 | 1.43 |
| ^a^ Exclude two sequences belonging to the Qinling haplogroup (see Fig. 3B) | | | |

**Clock rates of chloroplast DNA and ITS for *Taxus***

CpDNA and ITS c lock rates (u) were estimated using the formula: u = D/2T, where D and T stood for sequence divergence and divergence time, respectively, between *Taxus* and its sister monotypic genus species, *Pseudotaxus* *chienii*. We obtained the following T estimates from Liu *et al.* (2013) who reconstructed a dated phylogeny for 39 extant gymnosperm genera based on two cpDNA datasets and a total of 11 fossil calibration points: mean value = 93.3 Mya, 95% highest posterior density interval = 47.4–139.1 Mya.

To obtain D, we first compiled cp and ITS alignments for selected *Taxus* taxa alongside *P. chienii* (Table S5). We then estimated a D value for each *Taxus*-*Pseudotaxus* taxon pair using a maximum likelihood estimator in DIVEIN (Deng *et al.* 2010), with which we obtained an average D value over all comparisons. We specified nucleotide substitution models as GTR+G and HKY for cpDNA and ITS, respectively; both were selected in jModelTest 2.1.3 (Darriba *et al.*, 2012) based on the Bayesian information criterion (BIC). Resultant average D values were 0.113 and 0.207 substitutions per site for cpDNA and ITS, respectively. Based on the formula described above, we then obtained cpDNA and ITS mean clock rates (and 95% confidence sets) as 0.006 (0.004–0.012) and 0.011 (0.007–0.022) substitutions per site per 10 million years, respectively.

We incorporated these above clock rates into the MultiTypeTree (MTT) analyses using the following lognormal priors: log(mean) = -4.93, log(standard deviation) = 0.27 for cpDNA; log(mean) = -4.50, log(standard deviation) = 0.27 for ITS. We also included in the isolation with migration (IM) analysis the locus-wide estimates of these clock rates: 1.67E-6 (9.70E-7–2.62E-6) and 1.24E-6 (7.26E-7–1.89E-6) mutations per year for cpDNA and ITS, derived with locus lengths of 2321 and 1119 base pairs, respectively.

| **Table S5** GenBank accessions of *Taxus* and *Pseudotaxus* sequences used for deriving clock rates of chloroplast DNA and ITS. | | |
| --- | --- | --- |
| Taxon | cpDNA^a^ | ITS |
| *Taiwan T. phytonii* | PP394461 | PP388520 |
|  | PP394750 |  |
|  | PP395039 |  |
|  | PP395328 |  |
| *T. mairei* | PP394351 | PP388300 |
|  | PP394640 |  |
|  | PP394929 |  |
|  | PP395218 |  |
| Huangshan *Taxus* | PP394372 | PP388342 |
|  | PP394661 |  |
|  | PP394950 |  |
|  | PP395239 |  |
| *T. calcicola* | MH390461 | HM590953 |
| *T. chinensis* | MH390476 | HM590947 |
| *T. florinii* | MH390487 | HM590943 |
| Emei *Taxus* | MH390456 | HM590952 |
| Qinling *Taxus* | MH390444 | HM590961 |
| *P. chienii* | MH023407 | MT735121 |
| ^a^ *atp*I-H, *pet*G-*trn*P, *trn*H-*psb*A and *trn*L-F accession numbers are provided in consecutive rows for Taiwan *T. phytonii*, *T. mairei* and Huangshan *Taxus*; for other taxa, the accession numbers for the complete chloroplast genomes are given | | |

APPENDIX S2

**Figure S1** Distributions of (a) chloroplast and (b) ITS haplotypes over sampling sites (coded as in Fig. 1 of the main text).

**Figure S2** Marginal posterior distributions of the divergence time parameters (*t*) obtained from an IM analysis with data inputted (the upper plot) or only with priors (the lower plot). Parameters *t1* and *t2* denote divergence between Taiwan *T. phytonii* and Philippine *T. phytonii* and between *T. mairei* and the common ancestor of the former two taxa, respectively.


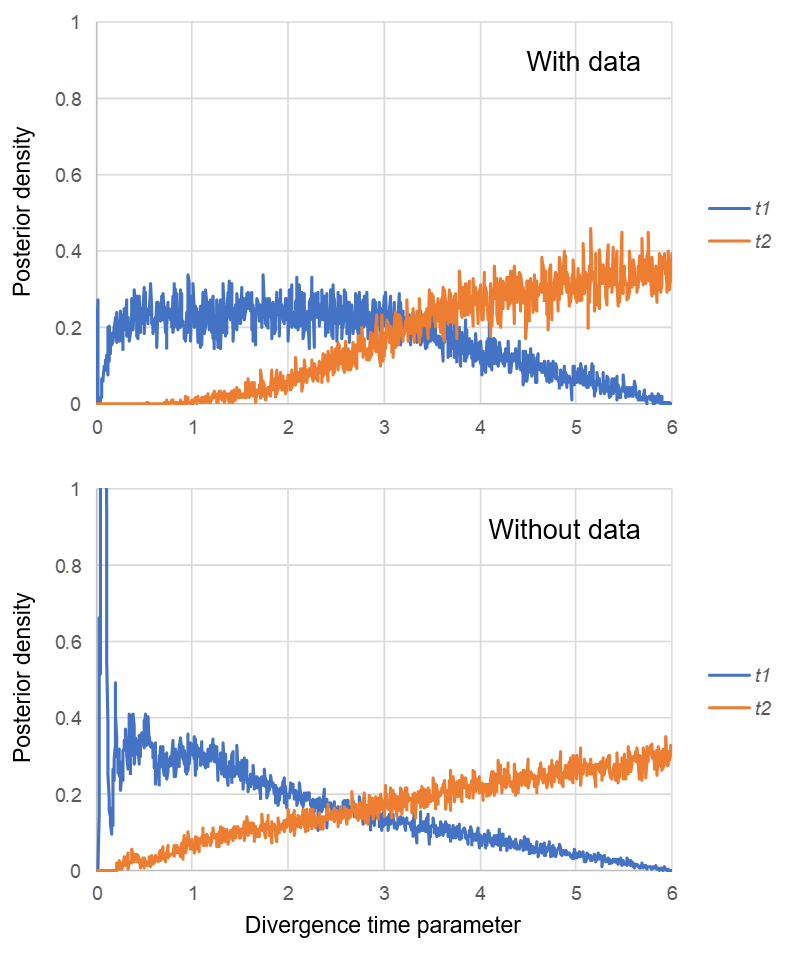


**Figure S3** Posterior estimations from the MultiTypeTree analyses for the numbers of *Taxus* lineage transferring events between Taiwan (TW) and the Philippines (PH). Blue bars denote the 95% confidence sets.


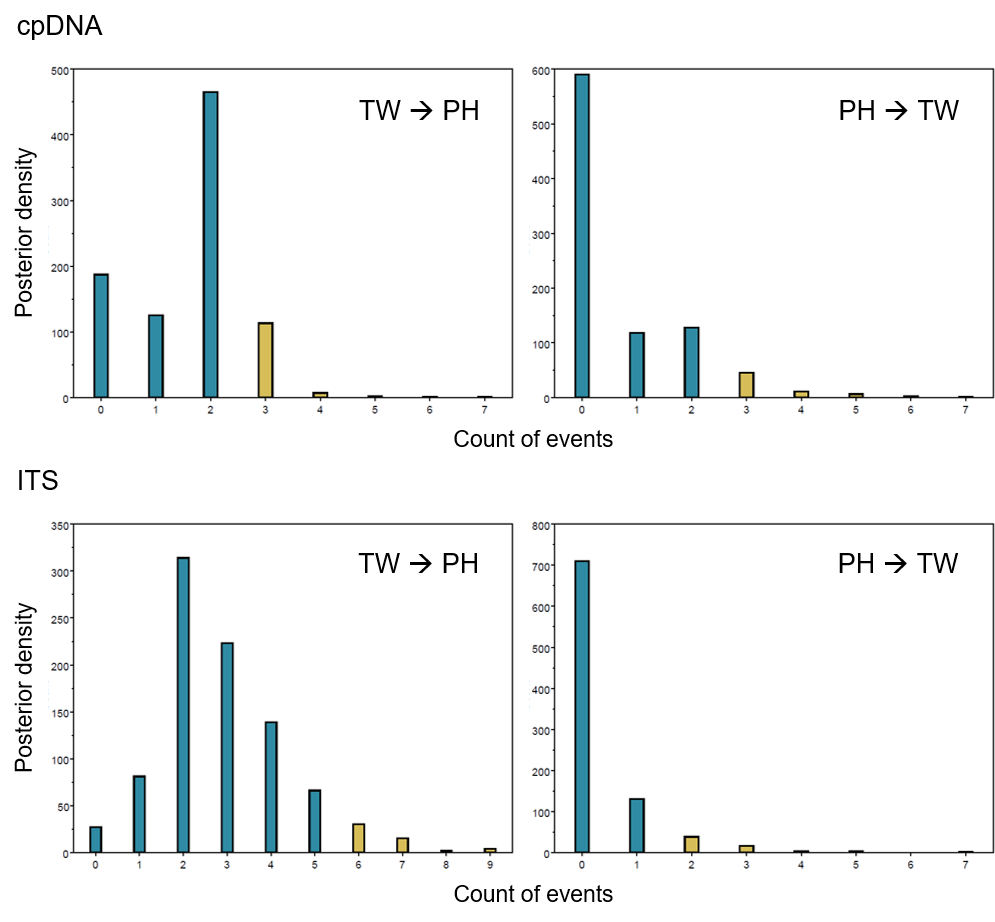


| **Table S6** Ages (million years ago) of selected tree nodes (#1–4) estimated from the MultiTypeTree analyses. For each marker type, values are shown in consecutive rows as means, lower 95% highest probability density limits (95HPDIs) and upper 95HPDIs. See Fig. 4 of the main text for node positions in the gene trees. | | | | |
| --- | --- | --- | --- | --- |
| Marker | #1 | #2 | #3 | #4 |
| cpDNA | 0.769 | 0.129 | 0.246 | 0.027 |
|  | 0.122 | 0.001 | 0.010 | <0.001 |
|  | 1.737 | 0.390 | 0.728 | 0.146 |
| ITS | 2.491 | 1.076 | 0.572 | 0.323 |
|  | 0.774 | 0.154 | 0.069 | 0.008 |
|  | 4.787 | 2.492 | 1.293 | 0.932 |

APPENDIX S3

**Introgression levels at chloroplast and biparental nuclear loci for *Taxus***

For *Taxus* (as well as for other dioecious plants each having cpDNA paternally inherited), we derive introgression levels at chloroplast (cp) and biparental nuclear (nr) loci under circumstances where unidirectional introgression has been carried out via either pollen dispersal alone or seed dispersal alone. First consider circumstances where pollen dispersal alone is in charged. Let *M*_p_ be the per generation proportion of the pollen pool for the ‘local’ species that is contributed from the ‘exotic’ one. In the local species, the proportion of introgressed cp genes (*I*_cp_) in generation t would be related to that in conspecific males (*I*_cp-m_) in generation t-1 by equation (1).

*I*_cp(t)_ = *M*_p_ + (1-*M*_p_)×*I*_cp-m(t-1)_ ……………………………………………………… (1)

In this equation, the first term on the right-hand side of the equal sign accounts for individual plants fathered by males of the exotic species, and the second term accounts for those fathered by conspecific males each carrying an earlier introgressed cp gene. In fact, in each generation the cp introgression levels would be the same in males and in females, assuming no differences among pollens of any kind (local vs. exotic pollen; male-determining vs. female-determining pollen) in fertilizing the seeds. Expressed in equations, *I*_cp-m(t)_ = [N*m*×*M*_p_ + N*m*×(1-*M*_p_)×*I*_cp-m(t-1)_]/N*m* and *I*_cp-f(t)_ = [N*f*×*M*_p_ + N*f*×(1-*M*_p_)×*I*_cp-m(t-1)_]/N*f*, where *m* and *f*, respectively, are proportions of males and females of the local species with a total population size of N. This renders *I*_cp-m(t)_ = *I*_cp-f(t)_ = *M*_p_ + (1-*M*_p_)×*I*_cp-m(t-1)_. Accordingly, we can simplify equation (1) as equation (2).

*I*_cp(t)_ = *M*_p_ + (1-*M*_p_)×*I*_cp(t-1)_ ………………………………………………………… (2)

Now consider the proportion of introgressed nr genes (*I*_nr_) in the local species under the influence of *M*_p_. Such an introgression level in generation t would be related to those in conspecific males and females (*I*_nr-m_ and *I*_nr-f_, respectively) in generation t-1 by equation (3).

*I*_nr(t)_ = 0.5×[*M*_p_ + (1-*M*_p_)×*I*_nr-m(t-1)_] + 0.5×*I*_nr-f(t-1)_ ………………………………….. (3)

Here the first term on the right-hand side of the equal sign accounts for chromosomes of individual plants obtained from their fathers (via pollen from either the same or different species), and the second term accounts for those obtained from their mothers (via ovules from conspecific females), each of which contributes to a half of the total genetic composition of any individual plant. Given that each seed to give rise to an individual plant of the local species, regardless of its sex, has its one chromosome the same likely sourced from a pollen of any kind and also has its second chromosome the same likely sourced from an ovule of any kind, we derive *I*_nr-m(t)_ = *I*_nr-f(t)_ for any generation t. It follows that equation (3) can be simplified as equation (4).

*I*_nr(t)_ = 0.5×[*M*_p_ + (1-*M*_p_)×*I*_nr(t-1)_] + 0.5×*I*_nr(t-1)_ ……………………………………... (4)

Using equations (2) and (4), we show in Figure S4 accumulations of *I*_cp_ and *I*_nr_ over generations under the effect of *M*_p_, respectively. This reveals a constantly higher *I*_cp_ than *I*_nr_ in each specific generation since the pollen dispersal across species started.

**Figure S4** An imaginary case showing introgression levels at chloroplast (cp) and nuclear (nr) loci in a dioecious plant species with cpDNA paternally inherited when this species receives genes from a second one via pollen dispersal. We assume in this case a constant per generation pollen dispersal rate (*M*_p_) = 0.0001 to give rise to individual plants in generation 1 and ever since (i.e. cp and nr introgression levels in generation 0 are both zero).

Next, we consider unidirectional introgression via seed dispersal alone. Let *M*_s_ be the per generation proportion of the seeds in the territory of the local species sent from the exotic one. To account for the fact that seed can have different sexes and that the sex ratio can vary across species, we specify *m*_loc_ and *m*_exo_ as proportions of the seeds of the local and the exotic species as males, respectively (female proportions are *f*_loc_ = 1- *m*_loc_ and *f*_exo_ = 1 - *m*_exo_). The cp introgression level in the local species, *I*_cp_, would follow equation (5) where the first term on the right-hand side of the equal sign accounts for seeds from the exotic species in the focal generation, and the second term account for those of own species fathered by conspecific males in the last generation each carrying an introgressed cp gene.

*I*_cp(t)_ = *M*_s_ + (1-*M*_s_)×*I*_cp-m(t-1)_ ……………………………………………………… (5)

To calculate *I*_cp_ with this equation, we also need to know how *I*_cp-m_ would change from one generation to the next one. This would be:

*I*_cp-m(t)_ = [N*M*_s_×*m*_exo_ + (N*m*_loc_-N*M*s×*m*_exo_)×*I*_cp-m(t-1)_]/N*m*_loc_; N is the population size of the local species. On the right-hand side of the equal sign of this equation, the first term of the numerator counts the number of males from the exotic species, and the second term of the numerator counts the number of males from the local species each carrying an introgressed cp gene. After algebraic operations, we obtain equation (6).

*I*_cp-m(t)_ = *M*_s_×*m*_exo_/*m*_loc_ + (1-*M*_s_×*m*_exo_/*m*_loc_)×*I*_cp-m(t-1)_ ………………………………. (6)

Equation (7) shows the nr introgression level in the local species, *I*_nr_, in response to *M*_s_. Here the first term on the right-hand side of the equal sign accounts for seeds from the exotic species, and the second term accounts for those of own species each having a ‘pollen site’ of the two sites of a nr locus contributed by an introgressed nr gene in a conspecific male and/or having the ‘ovule site’ contributed by an introgressed nr gene in a conspecific female in the last generation; 0.5 in the second term stands for the fact that each introgressed nr gene would contribute to - once passed - a half of the total genetic composition of the offspring at that locus in the next generation.

*I*_nr(t)_ = *M*_s_ + 0.5×(1-*M*_s_)×(*I*_nr-m(t-1)_ + *I*_nr-f(t-1)_)………………………………………. (7)

The dynamics of the nr introgression level in males of the local species would be:

*I*_nr-m(t)_ = [N*M*_s_×*m*_exo_ + 0.5×(N*m*_loc_-N*M*s×*m*_exo_)×(*I*_nr-m(t-1)_ + *I*_nr-f(t-1)_)]/N*m*_loc_

After algebraic operations, we obtain equation (8).

*I*_nr-m(t)_ = *M*_s_×*m*_exo_/*m*_loc_ + 0.5×(1-*M*_s_×*m*_exo_/*m*_loc_)×(*I*_nr-m(t-1)_ + *I*_nr-f(t-1)_) ………………. (8)

Similarly, we derive equation (9) for the dynamics of the nr introgression level in females of the local species.

*I*_nr-f(t)_ = *M*_s_×*f*_exo_/*f*_loc_ + 0.5×(1-*M*_s_×*f*_exo_/*f*_loc_)×(*I*_nr-m(t-1)_ + *I*_nr-f(t-1)_) …………………... (9)

Using equations (5)–(6) and (7)–(9), we show in Figure S5 accumulations of *I*_cp_ and *I*_nr_ over generations under the effect of *M*_s_, respectively, providing different combinations of sex ratios in the seeds from the local versus in the exotic species. When the sex ratio is the same between the two species, *I*_cp_ and *I*_nr_ show the same accumulation rate (Fig. S5a). When there are proportionally more males and more females in the exotic species than in the local one, *I*_cp_ values would accumulate faster and slower compared to *I*_nr_ values, respectively (Fig. S5b and S5c, respectively).

Equations (1)–(9) derived above hold for each of the two species showing bidirectional introgression with each other once the introgression rates in both directions are low, so the chance that a cp/nr gene sent out earlier later introgresses back is negligible.

**Figure S5** Imaginary cases showing introgression levels at chloroplast (cp) and nuclear (nr) loci in a dioecious plant species with cpDNA paternally inherited when this species receives genes from a second one via seed dispersal. In each case, a constant per generation seed dispersal rate (*M*_s_) = 0.0001 is assumed to occur since generation 1 (cp and nr introgression levels in generation 0 are both zero). Cases (a), (b) and (c) present situations, respectively, when the male proportion of the local species (*m*_loc_) is the same as, lower than and higher than that of the exotic species (*m*_exo_).

**References**

Anderson ED, Owens JN (1999) Megagametophyte development, fertilization, and cytoplasmic inheritance in *Taxus brevifolia*. *International Journal of Plant Sciences* **160**, 459-469.

Baum DA, Small RL, Wendel JF (1998) Biogeography and floral evolution of baobabs (*Adansonia*, Bombacaceae) as inferred from multiple data sets. *Systematic Biology* **47**, 181-207.

China Plant BOL Group, Li D-Z, Gao L-M , *et al.* (2021) Comparative analysis of a large dataset indicates that internal transcribed spacer (ITS) should be incorporated into the core barcode for seed plants. *Proceedings of the National Academy of Sciences of the United States of America* **108**, 19641-19646.

Darriba D, Taboada GL, Doallo R, Posada D (2012) jModelTest 2: more models, new heuristics and parallel computing. *Nature Methods* **9**, 772-772.

Deng W, Maust BS, Nickle DC*, et al.* (2010) DIVEIN: a web server to analyze phylogenies, sequence divergence, diversity, and informative sites. *Biotechniques* **48**, 405-408.

Dmitriev DA, Rakitov RA (2008) Decoding of superimposed traces produced by direct sequencing of heterozygous indels. *Plos Computational Biology* **4**, e1000113.

Flot J-F (2010) SeqPHASE: a web tool for interconverting phase input/output files and FASTA sequence alignments. *Molecular Ecology Resources* **10**, 162-166.

Flot J-F, Tillier A, Samadi S, Tillier S (2006) Phase determination from direct sequencing of length-variable DNA regions. *Molecular Ecology Notes* **6**, 627-630.

Fu C-N, Wu C-S, Ye L-J*,* *et al.* (2019) Prevalence of isomeric plastomes and effectiveness of plastome super-barcodes in yews (*Taxus*) worldwide. *Scientific Reports* **9**, 2773.

Hey J (2010) Isolation with migration models for more than two populations. *Molecular Biology and Evolution* **27**, 905-920.

Hey J (2011) Documentation for IMa2. Available from: https://bio.cst.temple.edu/~hey/program_files/IMa2/Using_IMa2_8_24_2011.pdf.

Hey J, Nielsen R (2007) Integration within the Felsenstein equation for improved Markov chain Monte Carlo methods in population genetics. *Proceedings of the National Academy of Sciences of the United States of America* **104**, 2785-2790.

Hwang L-H, Hwang S-Y, Lin T-P (2000) Low chloroplast DNA variation and population differentiation of *Chamaecyparis formosensis* and *Chamaecyparis taiwanensis*. *Taiwan Journal of Forest Science* **15**, 229-236.

Ji Y, Liu C, Landis JB, Deng M, Chen J (2021) Plastome phylogenomics of *Cephalotaxus* (Cephalotaxaceae) and allied genera. *Annals of Botany* **127**, 697-708.

Liu J, Möller M, Gao L-M, Zhang D-Q, Li D-Z (2011) DNA barcoding for the discrimination of Eurasian yews (*Taxus* L., Taxaceae) and the discovery of cryptic species. *Molecular Ecology Resources* **11**, 89-100.

Liu J, Möller M, Provan J, Gao L-M, Poudel RC, Li D-Z (2013) Geological and ecological factors drive cryptic speciation of yews in a biodiversity hotspot. *New Phytologist* **199**, 1093-1108.

Möller M, Liu J, Li Y*, et al.* (2020) Repeated intercontinental migrations and recurring hybridizations characterise the evolutionary history of yew (Taxus L.). *Molecular Phylogenetics and Evolution* **153**, 106952.

Sang T, Crawford DJ, Stuessy TF (1997) Chloroplast DNA phylogeny, reticulate evolution, and biogeography of *Paeonia* (Paeoniaceae). *American Journal of Botany* **84**, 1120-1136.

Shaw J, Lickey EB, Schilling EE, Small RL (2007) Comparison of whole chloroplast genome sequences to choose noncoding regions for phylogenetic studies in angiosperms: The tortoise and the hare III. *American Journal of Botany* **94**, 275-288.

Stephens M, Donnelly P (2003) A comparison of bayesian methods for haplotype reconstruction. *American Journal of Human Genetics* **73**, 1162-1169.

Stephens M, Smith NJ, Donnelly P (2001) A new statistical method for haplotype reconstruction from population data. *American Journal of Human Genetics* **68**, 978-989.

Taberlet P, Gielly L, Pautou G, Bouvet J (1991) Universal primers for amplification of three non-coding regions of chloroplast DNA. *Plant Molecular Biology* **17**, 1105-1109.

Tate JA, Simpson BB (2003) Paraphyly of *Tarasa* (Malvaceae) and diverse origins of the polyploid species. *Systematic Botany* **28**, 723-737.

Vaughan TG, Kühnert D, Popinga A, Welch D, Drummond AJ (2014) Efficient Bayesian inference under the structured coalescent. *Bioinformatics* **30**, 2272-2279.

Wang L-L, Shi Y-L, Wang C-X, Li X (2019) The complete chloroplast genome of the white-berry yew *Pseudotaxus chienii* (Cupressales: Taxaceae), a rare and endangered relict plant endemic to southern China. *Mitochondrial DNA Part B* **4**, 760-761.

Wang W-B, Jiang Y-B, Wang D-M, Zhou Y, Jing Y (2006) Biological and ecological characteristics of *Taxus yunnanensis*. *Journal of West China Forestry Science* **35**, 33-39.

Watterson GA (1975) On the number of segregating sites in genetical models without recombination. *Theoretical Population Biology* **7**, 256-276.

White TJ, Bruns T, Lee S, Taylor J (1990) Amplification and direct sequencing of fungal ribosomal RNA genes for phylogenetics. In: *PCR Protocols: A Guide to Methods and Applications* (eds. Innis MA, Gelfand DH, Sninsky JJ, White TJ), pp. 315-322. Academic Press, Inc., New York.
